# Supplementary material for: Magnetic-field-dependent quantum emission in hexagonal boron nitride at room temperature
Source: Nat Commun. 2019 Jan 15;10:222. doi: 10.1038/s41467-018-08185-8 (PMC6333818; doi:10.1038/s41467-018-08185-8)
Supplement: Supplementary file 1 — Supplementary Information [file 41467_2018_8185_MOESM1_ESM.pdf]

Supplementary Information for:

Magnetic-Field-Dependent Quantum Emission in Hexagonal Boron Nitride at Room Temperature

Exarhos *et al.*

I. SUPPLEMENTARY METHODS

A. Experimental Setup

Supplementary Figure 1 shows the general layout of the scanning confocal fluorescence microscope. We use a 0.9 NA objective (Olympus) and 592 nm continuous (CW) excitation with $175 - 550$ µW at the sample. The optics shown are used for all photoluminescence (PL) imaging, autocorrelation measurements, and PL spectra, with the exception of the polarizer in the collection line, which is only in place for emission polarization dependence measurements. Two single-photon counters are used for PL imaging and autocorrelation measurements: Excelitas (SPCM-AQRH-14-FC) and MPD (PDM-R) detectors. Photon autocorrelation measurements are performed using a Hanbury Brown-Twiss setup with a PicoQuant PicoHarp 300 time correlated single-photon counting module. PL spectra are obtained using a Princeton Instruments IsoPlane 160 spectrometer and PIXIS 100 CCD with a spectral resolution of 0.7 nm. Spectra are uncorrected for the wavelength-dependent transmission efficiency of the collection line.

Excitation polarization dependence is measured by rotating the linear polarization of the excitation laser using a half waveplate. The PL is not polarization-selected; all emitted PL ($\lambda_{PL} > 633$ nm), regardless of polarization, is collected. For the emission polarization measurements, the excitation polarization is fixed (typically at the angle which maximizes the collected PL for the defect in question) and a linear polarizer is placed in front of the detector and rotated to the desired emission polarization angles. Polarized emission with wavelengths between ∼710 nm and ∼745 nm is measured. Both the excitation and collection lines are corrected to account for the birefringence of the dichroic mirror and other optics in the microscope.

B. Sample Preparation

Single-crystal h-BN purchased from HQ Graphene is exfoliated onto patterned silicon wafers topped with a 90-nm-thick thermal SiO_2 layer as described in Ref. 1. Following exfoliation, samples undergo an O_2 plasma clean in an oxygen barrel asher (Anatech SCE 108) and are annealed in Ar at 850° for 30 minutes. Subsequently, the samples were imaged in a scanning electron microscope (SEM) operating at 3 kV (FEI Strata DB235 FIB SEM). The flake studied in this work was imaged for < 5 minutes, and the sample was in the SEM chamber for <30 minutes. Following

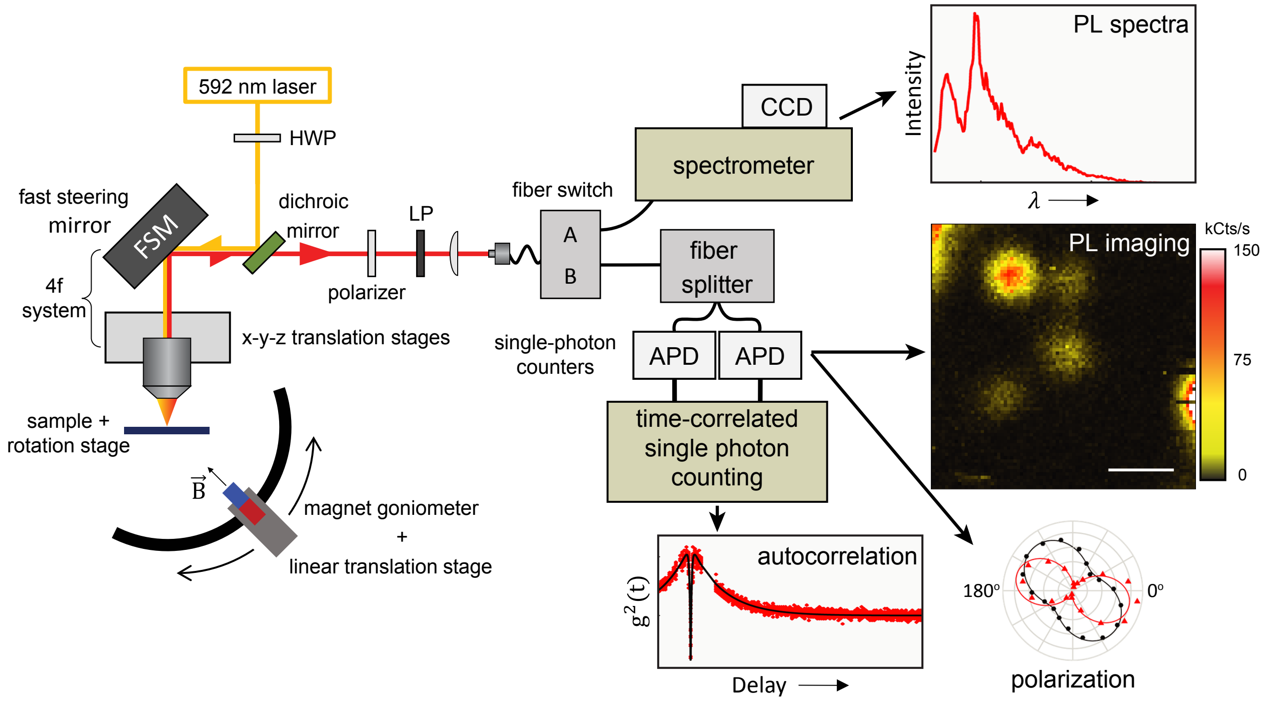

Supplementary Figure 1. **Scanning confocal fluorescence microscope.** Experimental setup for studying quantum emitters in h-BN. Abbreviations: HWP - half wave plate, LP - long pass filter, APD - avalanche photodiode. Scale bar denotes 0.5 µm. Figure adapted with permission from Ref. 1, copyright 2017 American Chemical Society.

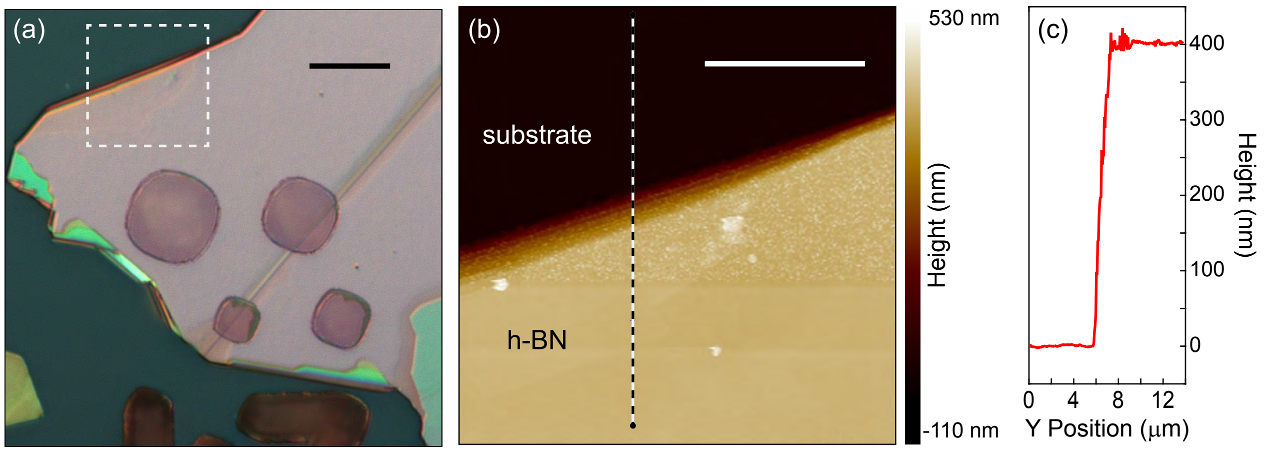

Supplementary Figure 2. **Optical and AFM characterization.** (a) Optical image of the h-BN flake. Scale bar corresponds to 10 μm. (b) AFM image of the h-BN flake. Scale bar corresponds to 5 μm. The region denoted by the dotted line in (a) corresponds to the area of the AFM scan shown in (b). (c) Line cut through the AFM scan at the dotted line in (b) showing the height of the h-BN flake.

SEM, the samples are again annealed in Ar at 850° for 30 minutes.

Supplementary Figure 2 shows a white light optical microscope image of the h-BN flake studied along with an atomic force microscope (AFM) image taken over a portion of the supported sample near the suspended region under study (dotted box in the optical image). A line cut through the AFM data shows that the sample is $\sim$ 400 nm thick.

C. Power Dependence of Magnetic Field-Dependent Emission

Supplementary Figure 3 shows the PL variation of the emitter studied in the main text due to an in-plane magnetic field as a function of the optical excitation power at 240 G. The variation appears to be independent of power across the range of settings used in our experiments.

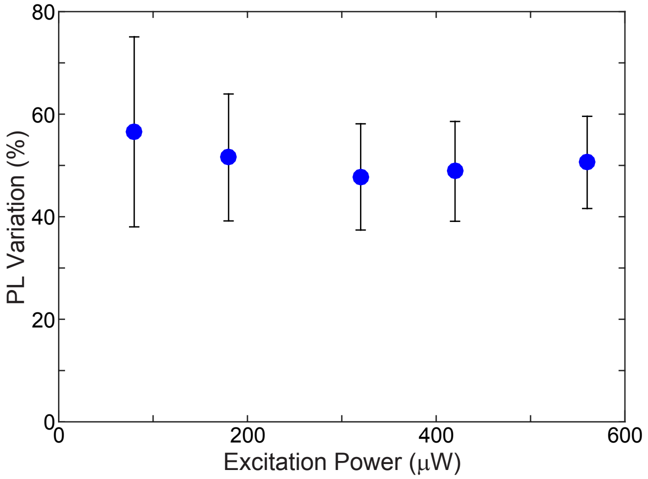

Supplementary Figure 3. **Power dependence of PL variation.** PL variation % as a function of excitation power for the main defect from the manuscript. The absorptive dipole is oriented parallel to the applied 240 G magnetic field. Error bars represent the variance of the background-subtracted time-averaged emission rate from three 60 s scans, each taken with and without the applied magnetic field at a particular power.

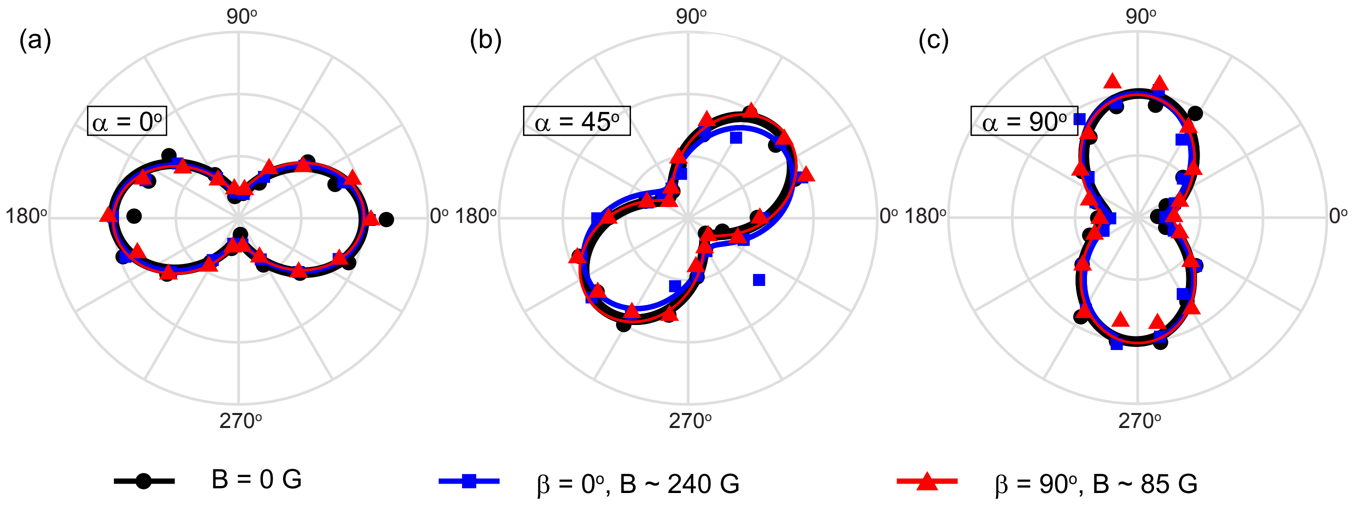

Supplementary Figure 4. **Absorptive optical dipole orientation field dependence.** Background-subtracted and normalized PL as a function of excitation polarization axes with fits to the absorptive dipole orientation for zero, in-plane, and out-of-plane applied magnetic fields at (a) $\alpha = 0°$, (b) $\alpha = 45°$, and (c) $\alpha = 90°$.

D. Field Independence of the Absorptive Dipole Orientation

The apparent orientation of the absorptive dipole for the defect featured in Figures 2-3 of the main text is unaffected by an applied magnetic field, as shown in Supplementary Figure 4. Excitation polarization scans taken at three different sample orientations ($\alpha = 0°$, 45°, and 90°) with applied magnetic fields both in-plane and out-of-plane display a fixed absorptive dipole orientation.

E. Analysis and Fits of Photon Counting Statistics

Photon autocorrelation data acquired for different settings of the applied magnetic field are fitted using an empirical model as described in the main text and corrected for the measured background. Best-fit parameters are listed in Supplementary Table 1.

Supplementary Table 1. **Magnetic-Field-Dependent Photon Emission Statistics**. Best-fit parameters to photon autocorrelation curves from the emitter reported in the main text at different orientations of an applied magnetic field.

(α, β)	C_1	C_2	C_3	τ_1 (ns)†	τ_2 (μs)	τ_3 (μs)	$\tilde{C}_1^\ddagger$	$\tilde{C}_2^\ddagger$	$\tilde{C}_3^\ddagger$
(0°,-)*	1.58±0.02	1.7±0.5	0.09±0.01	1.2±0.7	1.48±0.03	16±2	$5.6^{+0.8}_{-0.6}$	$6.0^{+0.9}_{-1.2}$	0.33±0.04
(45°,-)*	1.48±0.03	1.5±0.6	0.08±0.01	1.1±0.8	1.33±0.04	17±2	$5.4^{+0.8}_{-0.6}$	$5.3^{+1.3}_{-1.8}$	0.28±0.01
(0°,0°)	1.31±0.02	1.3±0.6	-	1.5±1.1	1.43±0.03	-	$3.00^{+0.14}_{-0.13}$	$2.9^{+1.0}_{-1.1}$	-
(45°,0°)	1.65±0.02	2.1±0.6	-	0.8±0.4	1.41±0.02	-	$6.0^{+0.9}_{-0.7}$	$7.7^{+1.0}_{-1.3}$	-
(90°,0°)	1.36±0.04	1.5±1.1	-	0.9±1.1	1.21±0.04	-	$3.26^{+0.16}_{-0.15}$	4±2	-

*$(\alpha, \beta) = (\alpha, -)$ indicates no applied magnetic field. When applied, the field strength is 240 G.
† τ_1 lifetimes are likely limited by detector timing jitter.
‡ Background corrected bunching amplitudes

F. Additional Magnetic-Field-Dependent Emitters

We observed reproducible PL variations in response to in-plane magnetic fields for a handful of other emitters in the same suspended region of the h-BN flake. For example, the emitter marked with a square in Fig. 1(b,c) of the main text exhibited a $\approx -20\%$ PL variation (Supplementary Figure 5) in the orientation shown. Other spots brightened in response to a field. Unfortunately, many of these emitters bleached away upon further study. Blinking

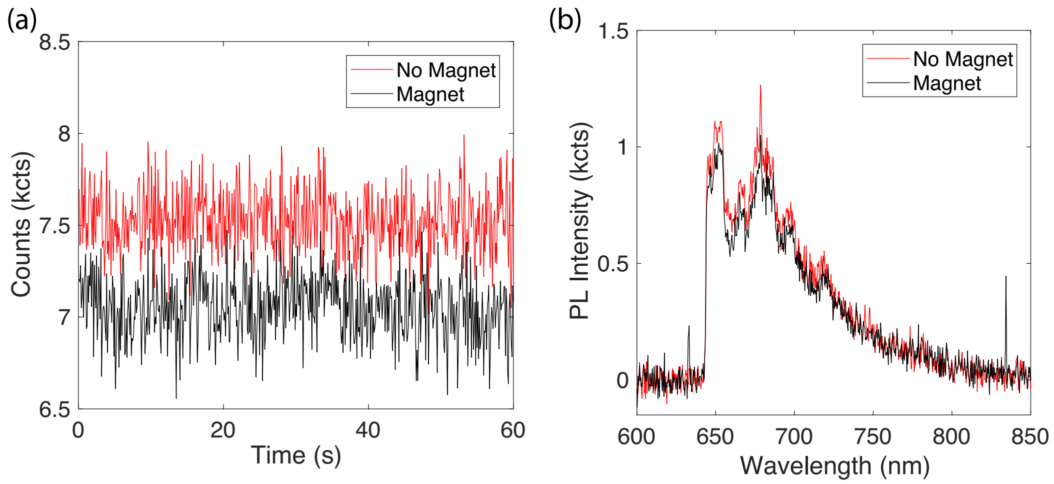

Supplementary Figure 5. **Magnetic field dependence of emitter marked square in main text.** (a) Emission in absence and presence of magnetic field averaged over three scans showing reduced emission when magnetic field is applied. (b) PL spectra showing reduced emission when magnetic field is applied.

and other instabilities on long timescales also complicate the field-dependent measurements. We estimate that $<5\%$ of emitters observed in this sample exhibit a reproducible field dependence, although we note that our searches for field-dependent emitters via differential images were taken at one particular magnetic field orientation and could have excluded emitters for which the field orientation resulted in no PL variation. Nonetheless, the number of field-dependent emitters compared to all emitters is small.

Supplementary Figure 6 summarizes a series of measurements concerning a field-dependent emitter in a different region of the same suspended flake considered in the main text. Supplementary Figure 6(a) shows a PL image of a suspended part of the flake which contains the emitter (circled). The PL image is obtained with linearly polarized 592 nm illumination (linear polarization setting of 20° relative to the horizontal) that maximizes emission, with an applied in-plane magnetic field in the horizontal direction of 25 G. All measurements are performed with an illumination power of 130 µW (measured before the objective).

Supplementary Figure 6(b) shows the PL variation as defined in the main text, $(I_B - I_0)/I_0$, where I_B, I_0 refer to the PL intensity for an in-plane field of 890 G and 25 G, respectively. The circled emitter's PL intensity decreases by $\sim$40%. Supplementary Figure 6(c) shows the emitter's PL spectrum with an applied in-plane magnetic field of 25 G and 890 G. The main component of the low-field PL spectrum between $\approx$700-800 nm clearly reflects the field-dependent decrease in emission rate, although interestingly there is a blue-shifted increase in PL around 670 nm when the field is applied. The field-independent feature around 650 nm is associated with the background. The peak at $\sim$730 nm at low field (25 G) as well as most emission in the range 700 nm - 750 nm is similar to that of the emitter discussed in main text. However, we note that other field-dependent emitters observed in this sample (e.g., the emitter whose spectrum is plotted in Supplementary Figure 5) exhibit qualitatively different spectra.

Supplementary Figure 6(d) shows the absorptive dipole orientation at different sample orientations for 25 G and 890 G in-plane magnetic field. The apparent dipole orientation is independent of applied magnetic field, similar to the emitter presented in the main text (see Supplementary Figure 4). Supplementary Figure 6(e) depicts the PL variation as a function of sample orientation, comparing in-plane magnetic field strengths of 25 G and 890 G. In contrast to the 90°-periodic pattern of brightening and dimming observed for the emitter studied in the main text, this defect exhibits reduced PL for all in-plane field directions. The dashed line signifies the average PL variation of $\sim$38%. Blinking is observed especially at low fields, and is responsible for the large spread in observations. The PL variation in response to an out-of-plane field (not shown) is also negative.

Supplementary Figure 6(f) depicts the photon autocorrelation function for in-plane magnetic field strengths of 25 G and 400 G. At both fields, the defect exhibits antibunching with a timescale, $\tau_1 \approx 7$ ns, and bunching over longer times. The data is fitted with the empirical model discussed in the text. The inset shows the photon autocorrelation data for 25 G, after correction for a Poissonian background (measured at a nearby location on the flake) and binned by delay on a linear scale. A fit to the data using a single exponential antibunching decay drops below the single-emitter criterion with a best-fit minimum value $\tilde{g}^{(2)} = 0.00 \pm 0.13 < 0.5$.

At low fields, at least three bunching decay terms are required to achieve a suitable fit to the data (i.e., $n = 4$ in the model), with characteristic bunching timescales $\tau_2 \approx 350$ ns, $\tau_3 \approx 180$ µs, and $\tau_3 \approx 2$ ms. When the field is

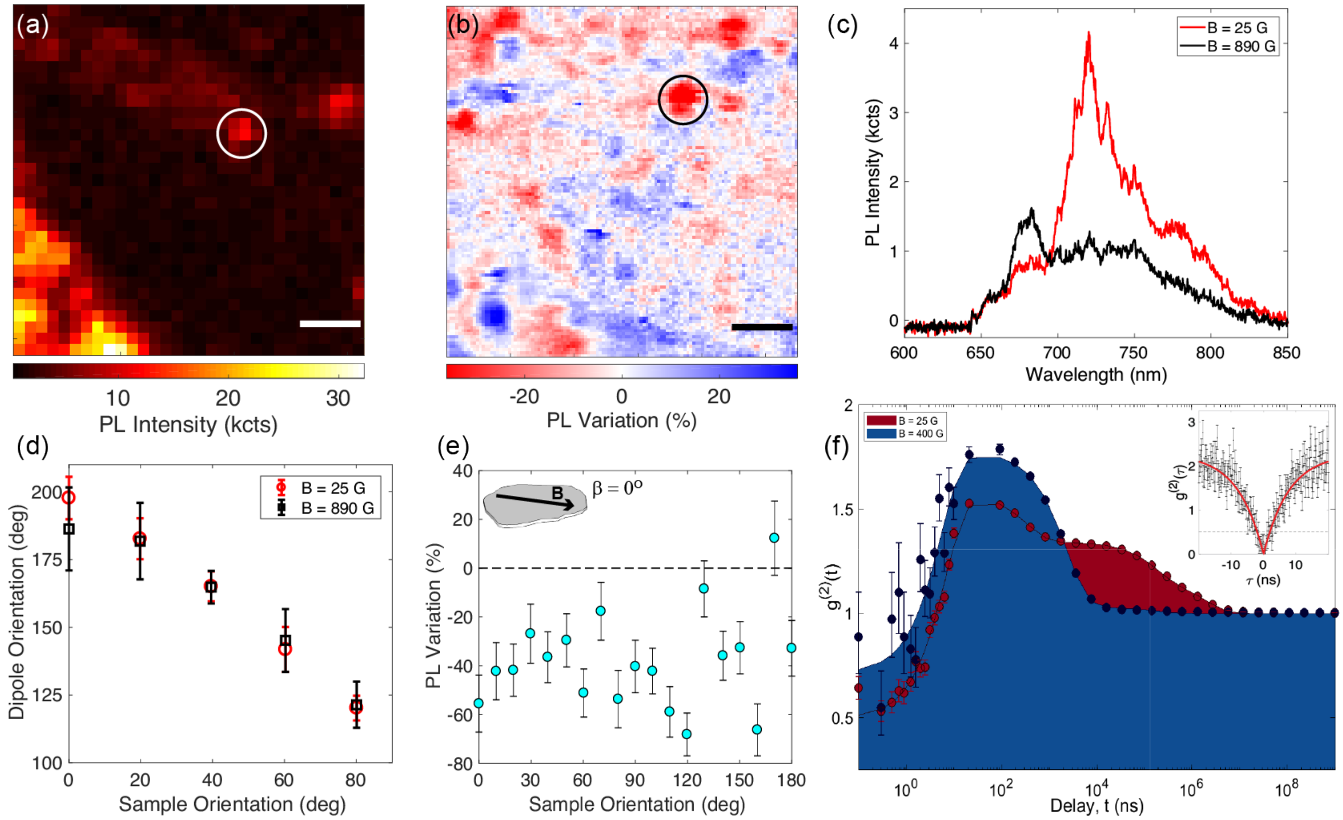

Supplementary Figure 6. **Additional magnetic field-dependent quantum emitter** (a) PL image of another region on the suspended h-BN flake shown in Figure 1(a) of main text. The circled spot is the additional emitter that showed magnetic field-dependent PL. Scale bar denotes 1 µm. (b) Background-subtracted differential PL variation image from the same region shown in (a) identifying changes due to an applied in-plane magnetic field. Circled red spot is the same region as the circle in (a). Scale bar denotes 1 µm. (c) PL spectra for the defect circled in (a,b) for in-plane magnetic fields of 25 G and 890 G, oriented such that **B** is parallel to the absorptive dipole. (d) Measured absorptive dipole orientation for 25 G and 890 G as a function of sample orientation. Error bars represent 95% confidence intervals from fits to excitation-polarization-dependent PL data using a dipole radiation sinusoidal function. (e) PL variation comparing 890 G and 25 G for various sample orientations. The dashed line shows the average PL variation. Error bars represent 68% confidence intervals derived from fits of two-dimensional Gaussian functions to spatial PL maps at different orientations and magnetic field settings. Each point is based on a pair of images with the field applied or absent. (f) Measured photon autocorrelation function (points) for in-plane applied magnetic fields ($\beta = 0°$) of 25 G and 400 G. Fits (black curves) are described in the text. The inset shows the background-corrected short-delay photon autocorrelation function. Error bars represent the Poissonian uncertainty based on photon counts in each bin. The dashed line shows the single photon emission criterion.

applied, the behavior is well-approximated by a single decay at $\tau_2 \approx 2.3$ µs. Again, this is qualitatively similar to the behavior of the defect in the main text, in that the long-timescale bunching vanishes with the field is applied, but it also is quantitatively different in several important respects. We believe that these differences can be captured within our model — even using the same basic level structures — through variations in the local defect parameters due, e.g., to strain that shifts the triplet zero-field splitting terms, variations in inter-system crossing (ISC) rates due to energy offsets between the triplet and singlet states, and variations in the triplet spin-T_1 relaxation time. Clearly, further investigation is required to understand the relevant perturbations and the range of possible field-dependent behaviors for different emitters.

G. Molecular orbital theory for h-BN defects and optical dynamics

The goal of our theoretical study is to use molecular orbital (MO) theory to enumerate a set of simplified models for defect electronic structure based on symmetry considerations [2], and then to perform semiclassical calculations to simulate their optical and spin dynamics under steady-state illumination, for comparisons with experimental results.

Supplementary Table 2. **Character table for C_{2v}**

	**E**	$\mathbf{C_2}$	$\sigma_\mathbf{v}$**(xy)**	$\sigma_\mathbf{v}$**(xz)**	**Linear functions**	**Quadratic functions**
A_1	1	1	1	1	x	x^2, y^2, z^2, S_x^2, S_y^2, S_z^2
A_2	1	1	-1	-1	S_x	yz, S_yS_z
B_1	1	-1	1	-1	y, S_z	xy, S_xS_y
B_2	1	-1	-1	1	z, S_y	xz, S_xS_z
$E^*_{1/2}$	2	0	0	2		

*Double group representation.

Supplementary Table 3. **Group multiplication table**

A_1	A_2	B_1	B_2	
A_1 ($\hat{X}$)*	A_2 (-)	B_1 ($\hat{Y}$)	B_2 ($\hat{Z}$)	A_1
	A_1 ($\hat{X}$)	B_2 ($\hat{Z}$)	B_1 ($\hat{Y}$)	A_2
		A_1 ($\hat{X}$)	A_2 (-)	B_1
			A_1 ($\hat{X}$)	B_2

*Optical selection rules shown in parentheses.

To that end, we do not start with a particular defect model and study it in detail; rather we explore the qualitative similarities and differences between various electronic configurations in an effort to narrow the space of possibilities. We hope this will motivate future efforts to compare these qualitative predictions with quantitative, *ab initio*, calculations of prospective defect configurations in h-BN.

1. *Choice of Point Group*

The starting point for any MO calculation is the identification of the relevant point group describing the symmetry of the molecule or defect system. Here, we focus on the point group C_{2v} based on the following considerations:

1. We universally observe optical selection rules in absorption and emission for linearly-polarized photons in the (x, y) plane, i.e., parallel to the hBN membrane. These selection rules naturally result from the symmetry-allowed orbitals in C_{2v} as shown below.

2. Our observations of field-dependent PL are consistent with underlying reflection symmetry about the (x, y) plane, whereas we observe fourfold (90°) rotational symmetry for in-plane fields.

3. C_{2v} symmetry is the expected point group for many defect complexes in h-BN, including distorted vacancies such as N_BV_N, and vacancy-impurity complexes such as C_BV_N, both of which have been proposed as models for hBN's visible quantum emission.

We view (1) and (2) as the most important considerations, and briefly discuss later why other possible point groups such as D_{3h} or C_s are unlikely to yield behavior consistent with our observations.

Details for the point group C_{2v} and its irreducible representations (IRs) are provided in Supplementary Table 2 and Supplementary Table 3. Note that we choose a coordinate system with x as the principal symmetry axis, lying in the h-BN plane, with the z-axis oriented normal to the h-BN plane.

2. Spin Hamiltonian

To begin, we consider only the electronic degrees of freedom, *i.e.*, neglecting hyperfine coupling with nuclear spins. In this case, we start with the generalized Hamiltonian for an electronic configuration with total spin S:

$$\begin{aligned} H &= \mu_{\rm B}\mathbf{S}\cdot g\cdot\mathbf{B} - \mathbf{S}\cdot\Lambda\cdot\mathbf{S} \\ &= \mu_{\rm B}(g_{xx}B_xS_x + g_{yy}B_yS_y + g_{zz}B_zS_z) + D\left(S_x^2 - \frac{1}{3}S(S+1)\right) + E(S_y^2 - S_z^2) \end{aligned} \tag{1}$$

Since spin-orbit coupling in h-BN is relatively weak, we assume that the components of the g-tensor are nearly equal to the bare value, $g \sim 2$, since spin-orbit corrections to the g-factor are of order $\frac{\lambda}{\Delta} \ll 1$, where λ is the spin-orbit strength and Δ is the orbital crystal field splitting. In C_{2v} we need to include two zero-field splitting (ZFS) parameters, D and E, in the fine structure term of H. This is in contrast to higher-symmetry cases such as C_{3v} or D_{3h} where E vanishes due to symmetry. The fact that E must be included is easy to see from the fact that all of the terms (S_x^2, S_y^2, S_z^2) transform like the trivial representation, A_1, and therefore are allowed to appear in the Hamiltonian, which also transforms as A_1 by definition. In our general treatment, D and E are empirical parameters; their origin can be either first-order spin-spin or second-order spin-orbit interactions, although spin-spin interactions are likely to dominate due to the weak spin-orbit coupling in h-BN. In this case, their values can be calculated explicitly in terms of two electron integrals given a specific orbital configuration [3–5]. Hence the Hamiltonian takes the simplified form:

$$H = g\mu_{\rm B}\mathbf{B}\cdot\mathbf{S} + D\left(S_x^2 - \frac{1}{3}S(S+1)\right) + E(S_y^2 - S_z^2) \tag{2}$$

We consider cases with total spin S = 0, $\frac{1}{2}$, 1, and $\frac{3}{2}$. Since there is no orbital degeneracy in C_{2v}, configurations with S$>\frac{1}{2}$ are likely to occur as metastable excited states, but they can also become the ground states in the case of closely-spaced orbitals and sufficient spin-exchange interactions, as predicted for some defects in hBN by Refs. [5–7]. However, states with higher spin require additional closely-spaced orbitals (*e.g.,* three orbitals in the case of a quartet) and therefore are progressively more unlikely. For this reason, we do not consider spin configurations with S$>\frac{3}{2}$.

We consider each case below:

S=0: Singlet configuration

Here the spin Hamiltonian vanishes. The symmetry of the spin-singlet configuration is A_1, as can be confirmed from the coupling coefficients and the double-group representation in C_{2v} (*e.g.* from Ref. [8]).

S=$\frac{1}{2}$: Doublet configuration

The ZFS vanishes in the spin doublet configuration, $H = g\mu_{\rm B}B\cdot\mathbf{S}$, where the components of $\mathbf{S}$ are 2×2 Pauli spin operators. The doublet components transform according to the double-group representation, $E_{1/2}$.

S=1: Triplet configuration

The zero-field spin eigenstates in the triplet configuration are non-degenerate due to the ZFS parameters. We can identify those eigenstates as the $\{|s_x\rangle, |s_y\rangle, |s_z\rangle\}$ spin basis

$$|s_x\rangle = \frac{1}{\sqrt{2}}(|\uparrow\downarrow\rangle + |\downarrow\uparrow\rangle) \sim A_2 \tag{3a}$$

$$|s_y\rangle = \frac{1}{\sqrt{2}}(|\uparrow\uparrow\rangle - |\downarrow\downarrow\rangle) \sim B_2 \tag{3b}$$

$$|s_z\rangle = \frac{1}{\sqrt{2}}(|\uparrow\uparrow\rangle + |\downarrow\downarrow\rangle) \sim B_1, \tag{3c}$$

where $|\uparrow\rangle$ and $|\downarrow\rangle$ are spin-1/2 eigenstates of S_x, and we determine the corresponding IR for each state using the symmetry coupling coefficients for the double group [8]. In this basis, the general Hamiltonian of Supplementary Equation 1 takes the form:

$$H = \begin{pmatrix} -\frac{2D}{3} & iB_zg_{zz}\mu_{\rm B} & B_yg_{yy}\mu_{\rm B} \\ -iB_zg_{zz}\mu_{\rm B} & \frac{D}{3} - E & B_xg_{xx}\mu_{\rm B} \\ B_yg_{yy}\mu_{\rm B} & B_xg_{xx}\mu_{\rm B} & \frac{D}{3} + E \end{pmatrix} \tag{4}$$

Here we notice that each Cartesian component of the magnetic field mixes one pair of spin eigenstates. In particular, in-plane components of the field mix $|s_x\rangle$ and $|s_y\rangle$ with $|s_z\rangle$, *i.e.*, in polar coordinates:

$$H|_{B_z=0} = \begin{pmatrix} -\frac{2D}{3} & 0 & g_{yy}\mu_\text{B} B\sin(\phi) \\ 0 & \frac{D}{3}-E & g_{xx}\mu_\text{B} B\cos(\phi) \\ g_{yy}\mu_\text{B} B\sin(\phi) & g_{xx}\mu_\text{B} B\cos(\phi) & \frac{D}{3}+E \end{pmatrix} \tag{5}$$

Such mixing is essential to our models of spin-dependent fluorescence, and the symmetry is important. In this case, the eigenstates exhibit underlying 180° rotational symmetry (they depend on the relative sign between the two mixing terms but not the overall sign). However, we will show below that in situations where the optical dynamics depend on the overall projection of the triplet eigenstates on $|s_z\rangle$, or on mixing between $|s_x\rangle$ and $|s_y\rangle$ (which for the case of in-plane fields can only occur through $|s_z\rangle$), the resulting fluorescence can exhibit 90° symmetry, consistent with our observations.

For example, Supplementary Figure 7 shows the spin eigenvalues and projections on each basis vector as a function of in-plane magnetic field direction, when $E = 0.2D$ and $g\mu_\text{B}B/D = 0.5$. Note that the overall pattern has 180° symmetry, but that the mixing between states dominated by $|s_x\rangle$ and $|s_y\rangle$ (blue and orange) exhibits 90 deg symmetry.

$S=\frac{3}{2}$: Quartet configuration

The quartet is split by the ZFS into two Kramers doublets at energies $\pm\sqrt{D^2+3E^2}$. The zero-field Hamiltonian in the x-projection basis, $m_s = \{\frac{3}{2}, \frac{1}{2}, -\frac{1}{2}, -\frac{3}{2}\}$ takes the form:

$$H = \begin{pmatrix} D & 0 & \sqrt{3}E & 0 \\ 0 & -D & 0 & \sqrt{3}E \\ \sqrt{3}E & 0 & -D & 0 \\ 0 & \sqrt{3}E & 0 & D \end{pmatrix} \tag{6}$$

Assuming D≫E, the doublets consist mostly of the m_s=$\pm\frac{1}{2}$ and $\pm\frac{3}{2}$ eigenstates, respectively, but the E term slightly mixes the pairs of states $\{\frac{3}{2}, -\frac{1}{2}\}$ and $\{\frac{1}{2}, -\frac{3}{2}\}$. This makes sense from a group theory perspective, since there is only one double group representation for C_{2v}, and so both doublets will transform under the same IR: $E_{1/2}$. It is also easy to show using the coupling coefficients in Ref. 8 that the combinations $|\uparrow\uparrow\uparrow\rangle \sim |\frac{3}{2}\rangle$ and $|\uparrow\downarrow\downarrow\rangle \sim |-\frac{1}{2}\rangle$ both transform like $E_{1/2}^{-1/2}$ whereas $|\downarrow\downarrow\downarrow\rangle \sim |-\frac{3}{2}\rangle$ and $|\uparrow\uparrow\downarrow\rangle \sim |\frac{1}{2}\rangle$ both transform like $E_{1/2}^{+1/2}$, which is consistent with the predicted mixing even at zero field.

Notably, however, the rotational symmetry in response to in-plane magnetic fields is different than for the triplet case. The Hamiltonian as a function of B in polar coordinates becomes:

$$H|_{B_z=0} = \begin{pmatrix} D+\frac{3}{2}B\cos[\phi]g_{xx}\mu_\text{B} & \frac{1}{2}\sqrt{3}B\sin[\phi]g_{yy}\mu_\text{B} & \sqrt{3}E & 0 \\ \frac{1}{2}\sqrt{3}B\sin[\phi]g_{yy}\mu_\text{B} & -D+\frac{1}{2}B\cos[\phi]g_{xx}\mu_\text{B} & B\sin[\phi]g_{yy}\mu_\text{B} & \sqrt{3}E \\ \sqrt{3}E & B\sin[\phi]g_{yy}\mu_\text{B} & -D-\frac{1}{2}B\cos[\phi]g_{xx}\mu_\text{B} & \frac{1}{2}\sqrt{3}B\sin[\phi]g_{yy}\mu_\text{B} \\ 0 & \sqrt{3}E & \frac{1}{2}\sqrt{3}Bsin[\phi]g_{yy}\mu_\text{B} & D-\frac{3}{2}Bcos[\phi]g_{xx}\mu_\text{B} \end{pmatrix} \tag{7}$$

Here, whereas the eigenvalues still exhibit 180° rotational symmetry, the eigenvectors exhibit 360° deg symmetry. Supplementary Figure 8 gives an example using the same parameters as for the triplet case in Supplementary Figure 7. There is no indication of couplings between the states that could give rise to dynamics with 90° symmetry in this case.

Even more importantly, we will explore below how spin-orbit interactions give rise to spin-dependent selection rules for the ISC between singlet and triplet states, which subsequently produce changes in the PL as a function of magnetic field orientation. For the case of the doublet-to-quartet ISC, however, such selection rules do not arise naturally on

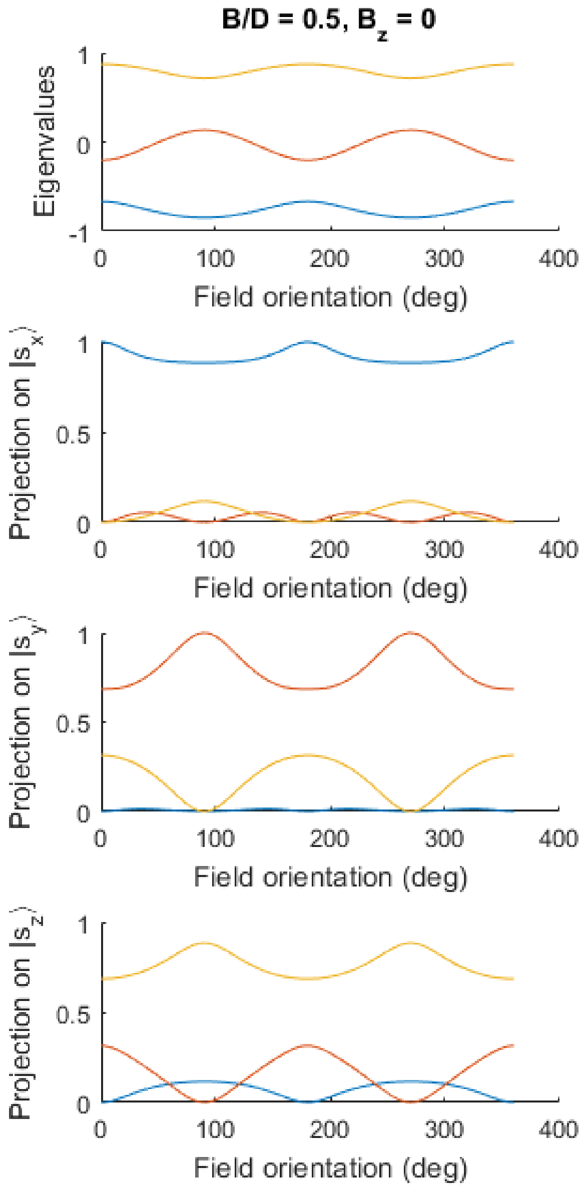

Supplementary Figure 7. **Spin-Triplet Eigenstates.** Energy eigenvalues (units of D) and corresponding projections on the $\{|s_x\rangle, |s_y\rangle, |s_z\rangle\}$ basis, as a function of in-plane orientation of an applied magnetic field.

the basis of symmetry, since there is only one double-group representation in C_{2v} that must describe all states in both manifolds. For these reasons, we believe it is unlikely that a defect with S=$\frac{1}{2}$ or S=$\frac{3}{2}$ configurations can be consistent with our observations, and we do not consider these systems further.

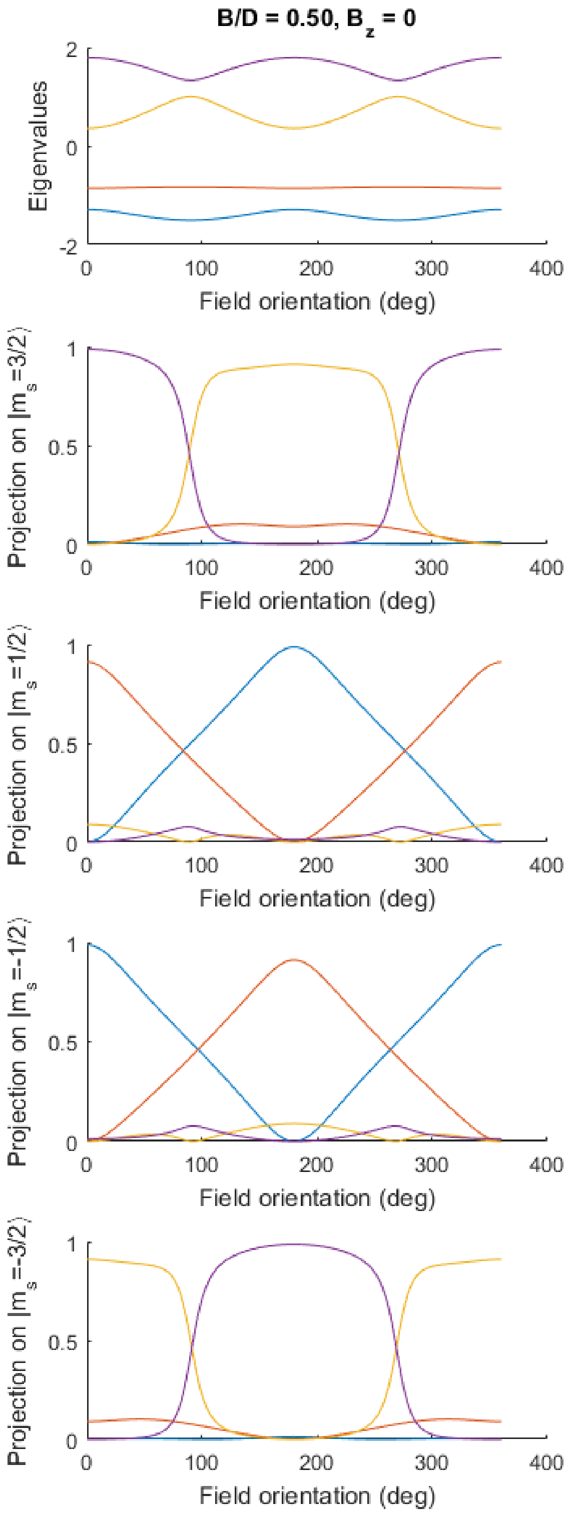

Supplementary Figure 8. **Spin-Quartet Eigenstates.** Energy eigenvalues (units of D) and corresponding projections on the S_x basis, $m_s = \{3/2, 1/2, -1/2, -3/2\}$, as a function of in-plane orientation of an applied magnetic field. Zero-field splitting parameters are the same as in Supplementary Figure 7.

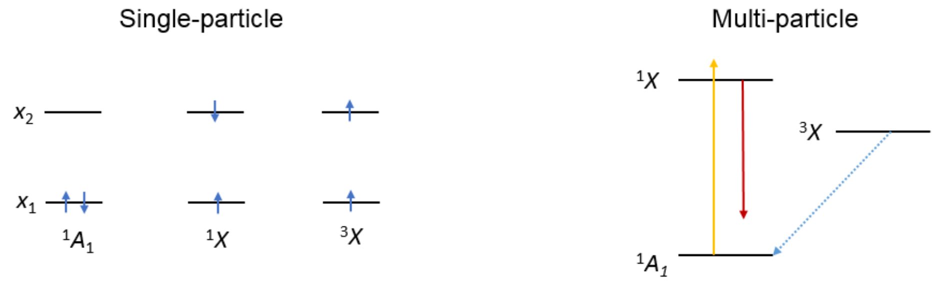

Supplementary Figure 9. **Electronic configurations (Singlet-GS).** Two single-particle levels of arbitrary symmetry x_1 and x_2 occupied by two electrons give rise to three multi-particle levels as shown at right.

3. Electronic Level Structure: Jablonski Diagrams

Following the MO theory treatment [2], we enumerate the configurations of single-particle and multi-particle energy levels that can arise from defects with C_{2v} symmetry, together with their radiative (optical dipole) and non-radiative (ISC) transition selection rules. Based on the considerations above, we consider cases including spin singlet and triplet manifolds, either of which could contain the ground state (GS) and fluorescent excited-state (ES).

Spin-singlet ground state:

The simplest case we could consider includes only two single-particle (SP) levels active in the optical dynamics, which can be arranged in a ground or excited-state singlet, or a triplet state with intermediate energy, as shown in Supplementary Figure 9. Based on the multiplication rules (Supplementary Table 3), we know that the ground state must have symmetry $^1A_1$ independent of the symmetry of the SP levels. The symmetry of the singlet ES and optical selection rules are similarly determined from the group multiplication table. In modeling the ISC, we assume the transition is mediated by spin-orbit coupling, with symmetric phonons accounting for the energy relaxation. The allowed transitions are those for which the total spin-orbit symmetry of the system is conserved. (This is the case, for example, for the ($^3E \rightarrow ^1 A_1$) ISC for the diamond NV center, whose spin-selectivity arises since there exists a combination of $m_s = \pm 1$ levels in ^3E that have spin-orbit symmetry A_1, forming an allowed transition to the singlet level which also transforms like A_1.) Therefore, it is helpful to enumerate the spin-orbit symmetry of each spin sublevel using the symmetrized spin-triplet states of Supplementary Equation (3) and the group multiplication rules (Supplementary Table 3). The resulting spin-orbit representations are listed in Supplementary Table 4:

Supplementary Table 4. **Spin-Orbit Representations**

**Orbital**	$\lvert S_x\rangle$	$\lvert S_y\rangle$	$\lvert S_z\rangle$
$^3A_1$	$\lvert A_2\rangle$	$\lvert B_2\rangle$	$\lvert B_1\rangle$
$^3A_2$	$\lvert A_1\rangle$	$\lvert B_1\rangle$	$\lvert B_2\rangle$
$^3B_1$	$\lvert B_2\rangle$	$\lvert A_2\rangle$	$\lvert A_1\rangle$
$^3B_2$	$\lvert B_1\rangle$	$\lvert A_1\rangle$	$\lvert A_2\rangle$

Using Supplementary Table 4, we can determine which spin eigenstate is allowed to pass through the ISC. Noting that the spin-orbit symmetry of all three triplet states is distinct from that of the orbital state alone, we observe that there are no spin-orbit-allowed transitions between the states ^1X and ^3X in Supplementary Figure 9. These transitions can be allowed via other means (e.g., through spin-spin interactions or asymmetric phonons) but are not likely to be strongly spin selective. Depending on the symmetry of X, there could be a spin-selective ISC back to the $^1A_1$ ground state.

In our experiments, we observe optical dipole transitions with in-plane linear polarization selection rules. There are only two possibilities for the multi-particle level structure that are consistent with this observation, as shown in Supplementary Figure 10. In the case of Supplementary Figure 10(a), the two SP orbitals are the same ($x_1 = x_2$), and there are no spin-orbit-allowed ISC transitions. We should not expect to see any spin-dependent effects in this situation. In Supplementary Figure 10(b), the two SP orbitals are different (chosen from either $\{A_1, B_1\}$ or $\{A_2, B_2\}$), and the metastable triplet decay should be spin dependent.

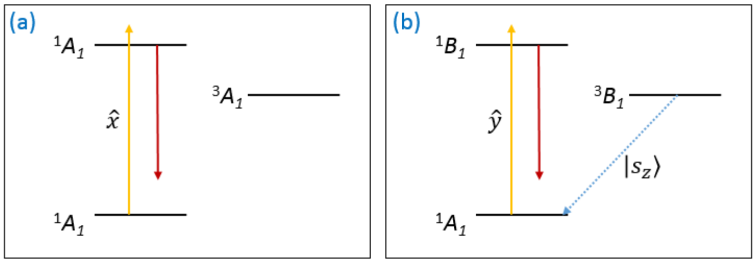

Supplementary Figure 10. **Jablonski Diagrams (Singlet-GS).** Level diagrams (a,b) represent specific configurations as in Supplementary Figure 9 consistent with in-plane optical dipole transitions.

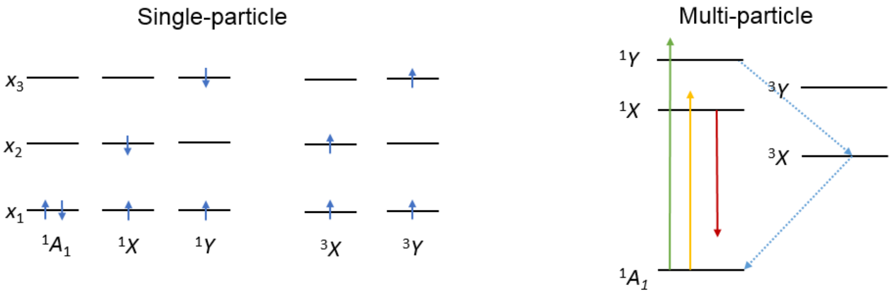

Supplementary Figure 11. **Additional excited states (Singlet-GS).** Generalized electronic configurations formed from three SP states and a singlet GS.

It is also possible that more than two SP levels are involved. In fact, we suspect additional excited states are likely to play a role based on the difference between the polarization dependence of the absorptive and emissive optical transitions. For example, the next-simplest level structure is shown in Supplementary Figure 11. Still, since the PL is strongly polarized in plane, the state ^1X is probably $^1A_1$ or $^1B_1$ as above, so the only qualitative change here might be that the brief occupation of the state ^1Y might contribute a spin-dependent pathway to the ^3X triplet state, which will be symmetry-allowed if $X \neq Y$. Depending on the level spacings, a further $^1A_1$ singlet excited state resulting from double occupation of the x_2 SP level might also be close in energy to ^1Y. However, any such states would be transiently occupied, most likely for a timescale shorter than the optical relaxation time ($\sim$1 ns) and therefore we ignore them hereafter from the point of view of simulating ISC dynamics.

Spin-triplet ground state:

For the case of a ground-state spin triplet, we need at least three SP levels to encompass the optical ground and excited states, as shown in Supplementary Figure 12. As before, the lowest-lying singlet state must have symmetry $^1A_1$. Since the orbital configurations X and Y can each be chosen from one of the four IRs in C_{2v}, this arrangement gives rise $4^2 = 16$ potential configurations of multi-particle states. But again, we can reduce this set by considering

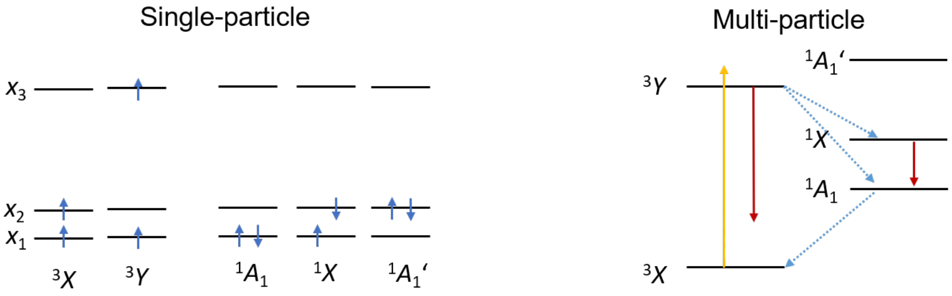

Supplementary Figure 12. **Electronic configurations (Triplet-GS).** Three single-particle levels of arbitrary symmetry x_1, x_2, x_3 occupied by two electrons give rise to multi-particle levels as shown at right.

only those configurations which exhibit an optical selection rule for in-plane polarization between ^{3}X and ^{3}Y. Based on the C_{2v} multiplication table, we see that:

- If the emission is polarized along x, then $X = Y$.
- If the emission is polarized along y, then $\{X, Y\} \in \{A_1, B_1\}$ or $\{A_2, B_2\}$.

This reduces the number of possible combinations from 16 to 8.

We also note that there will be singlet excited states that have orbital parts of the form $|x_1x_2\rangle + |x_2x_1\rangle$, and similarly for the x_1x_3 combination, whose total orbital symmetry will be the same as the triplet states X and Y, respectively. Especially if the states x_1 and x_2 are close in energy, as would be expected if exchange splitting is strong enough to make ^3X the ground state, we should expect another singlet state ^1X of the same symmetry to be close in energy to $^1A_1$, so it can also potentially contribute to the ISC. As shown in Supplementary Figure 12, an additional $^1A_1'$ state composed of the $(x_2)^2$ SP-level configuration could also play a role in the dynamics, although it is likely that Coulomb interactions will increase the energy spacing between two singlet levels of the same symmetry such as $^1A_1'$ and $^1A_1$. Finally, similarly to the case of the singlet-GS configurations in Supplementary Figure 11, additional triplet excited states could play a role in off-resonant absorption (e.g., the triplet state composed of the (x_2x_3) SP-level configuration), but we assume they do not contribute to the radiative or ISC relaxation pathways.

We account for all of these possibilities by varying the choice of ISC selection rules. As before, the spin-dependent ISC selection rules are determined by the spin-orbit-symmetrized triplet configuration (Supplementary Table 4) that transforms identically to the corresponding singlet state. We assume that optical-dipole allowed relaxation within the singlet levels means that the final ISC transition will always occur from the lowest-lying $^1A_1$ state. The 8 resulting Jablonski diagrams are listed in Supplementary Figure 13.

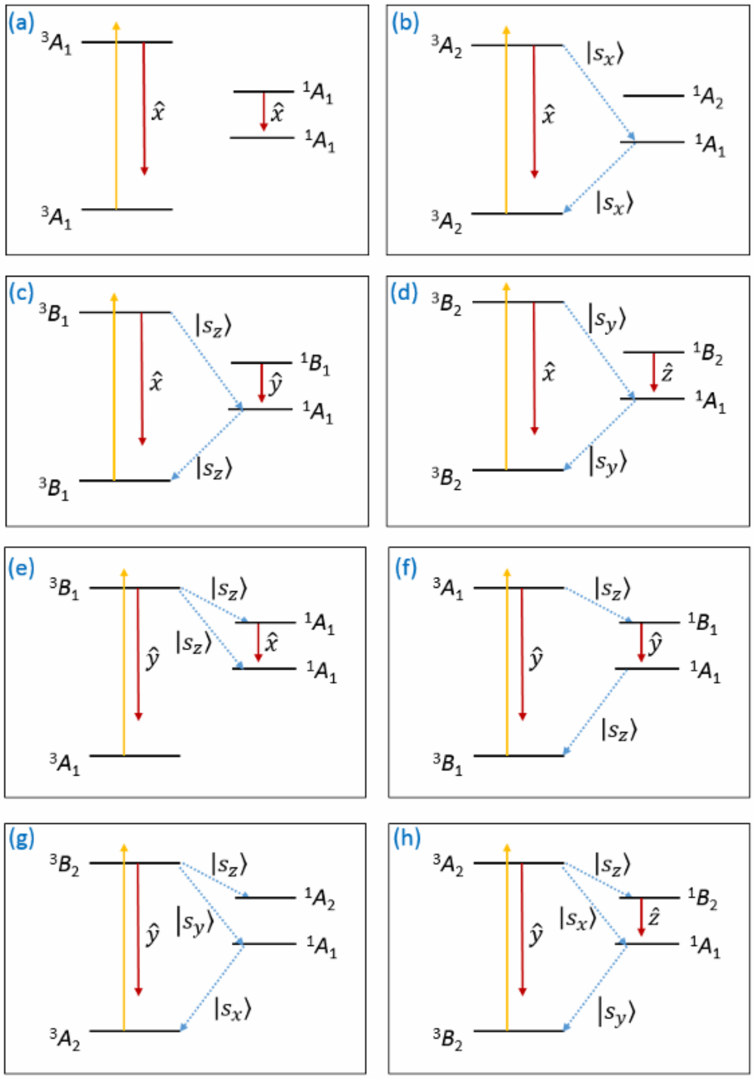

Supplementary Figure 13. **Jablonski Diagrams (Triplet-GS).** Level diagrams (a)-(h) represent specific configurations as in Supplementary Figure 12 consistent with in-plane optical dipole transitions.

4. Modeling Optical Dynamics:

We use a semiclassical Master Equation (ME) model to simulate the orbital and spin dynamics of these systems under optical illumination. For example, level structure (b) of the singlet-GS cases consists of five individual states, coupled by rates as indicated in Supplementary Figure 14. Here Γ_e is the optical excitation rate, Γ_s is the spontaneous radiative decay rate, and Γ_{ISC1}, Γ_{ISC2} are the non-radiative ISC rates to and from the metastable triplet state, respectively. The ISC spin selection rules are encapsulated in the coefficients m_i, $m_{i'}$ given by

$$m_i = \sum_{\mu} p_\mu |\langle s_\mu | s_i \rangle|^2, \tag{8}$$

where $\mu \in \{x, y, z\}$ and p_μ are the normalized selection rules between the singlet and the corresponding zero-field eigenstate $|s_\mu\rangle$. This incoherent sum over projections onto the field-dependent eigenstates $|s_i\rangle$ corresponds to the usual assumption that the ISC transitions are incoherent, *i.e.*, that the triplet state resulting from an ISC is described by a density matrix

$$\rho_{triplet} = m_1|s_1\rangle\langle s_1| + m_2|s_2\rangle\langle s_2| + m_3|s_3\rangle\langle s_3| \tag{9}$$

We can also include additional spin relaxation processes within the triplet state. With knowledge about a specific process (e.g., hyperfine or spin-phonon coupling) one could include decoherence using the Lindblad ME approach. Here, in order to capture the qualitative effects, we simply include spin relaxation through a set of uniform transition elements connecting all three pairs of triplet states, at the rate $1/T_1$. Hence the ME takes the form $\dot{\mathbf{x}} = R\mathbf{x}$, where the off-diagonal elements of the rate matrix are

$$R - \mathrm{diag}(R) = \begin{pmatrix} & \Gamma_s & m_3\Gamma_{\mathrm{ISC2}} & m_4\Gamma_{\mathrm{ISC2}} & m_5\Gamma_{\mathrm{ISC2}} \\ \Gamma_e & & 0 & 0 & 0 \\ 0 & m_{3'}\Gamma_{\mathrm{ISC1}} & & \frac{1}{T_1} & \frac{1}{T_1} \\ 0 & m_{4'}\Gamma_{\mathrm{ISC1}} & \frac{1}{T_1} & & \frac{1}{T_1} \\ 0 & m_{5'}\Gamma_{\mathrm{ISC1}} & \frac{1}{T_1} & \frac{1}{T_1} & \end{pmatrix} \tag{10}$$

and the diagonal components are simply $R_{ii} = -\Sigma_j R_{ji}$ in order to conserve total probability.

The ME for the triplet-GS cases are constructed in a similar way for a system with seven states as shown in Supplementary Figure 15. Here we additionally make the assumption that optical excitation and emission conserve the triplet spin state, ignoring spin-mixing transitions that might occur due to the fact that the ground and excited-state spin Hamiltonians are different. We also ignore spin relaxation in the triplet excited state due to its short (∼1 ns) lifetime.

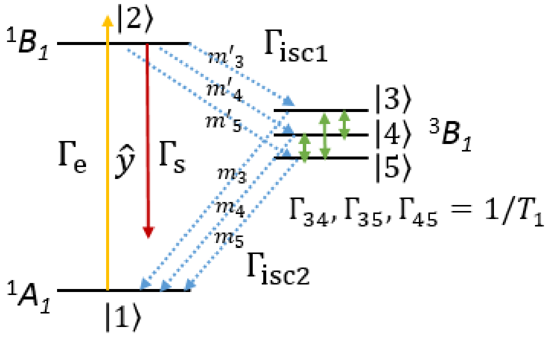

Supplementary Figure 14. **Transition Diagram (Singlet GS Model).** Rates and selection rules determine the structure of a master equation for the optical dynamics of singlet-GS models.

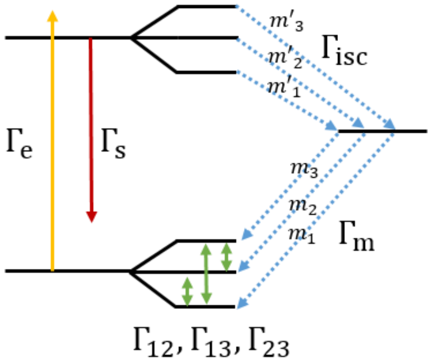

Supplementary Figure 15. **Transition Diagram (Triplet GS Model).** Rates and selection rules determine the structure of a master equation for the optical dynamics of triplet-GS models.

Steady-state PL and the $g^{(2)}$ function:

Once the master equation is constructed for a given set of parameters and field settings, the steady-state PL is calculated from the solution of $\langle \dot{\mathbf{x}} \rangle = R\langle \mathbf{x} \rangle = \mathbf{0}$, *i.e.*, from the null space of R. In the general case, $\langle \mathrm{PL} \rangle = \sum_{i\in \mathrm{GS}, j\in \mathrm{ES}} R_{ij}\langle x_j \rangle$, which reduces to $\langle \mathrm{PL} \rangle = \Gamma_s \sum_{i\in \mathrm{ES}} \langle x_i \rangle$ in the case of spin-conserving emission.

The autocorrelation function, meanwhile, is calculated by numerically integrating the ME starting with an initial condition corresponding to the configuration that follows emission of a photon. For the singlet-GS case, this initial state is simply $x_{\mathrm{GS}}(t=0) = 1$, whereas for the triplet-GS case the initial population is distributed in the ground state according to

$$\mathbf{x}_{\mathrm{GS}}(0) = P \cdot \frac{\langle \mathbf{x}_{\mathrm{ES}} \rangle}{\sum \langle \mathbf{x}_{\mathrm{ES}} \rangle}, \tag{11}$$

where $P_{ij} = R_{ij}/\sum_i R_{ij}$ gives the branching ratio probabilities for decay from ES state j to GS state i. In the case where the transitions are spin conserving, $P = I$. [Note that, in general $\mathbf{x}_{\mathrm{GS}}(0) \neq \langle \mathbf{x}_{\mathrm{GS}} \rangle$]. Given this initial condition, the autocorrelation function is related to the subsequent evolution of the excited-state population *via*

$$g^{(2)}(t) = \frac{\mathrm{PL}(t)}{\langle \mathrm{PL} \rangle} = \frac{\sum_{i\in \mathrm{GS}, j\in \mathrm{ES}} R_{ij} x_j(t)}{\langle \mathrm{PL} \rangle} \tag{12}$$

Free parameters in simulations

The simulations require values for a number of parameters, some of which can be related directly to experimental observations whereas others are less constrained. The spin-triplet Hamiltonian depends on the zero-field splitting parameters D and E. For typical molecules and point defects, these parameters have values ranging from a few hundred megahertz to a few gigahertz ($\sim$1-10 μeV in energy units). Therefore, in simulations we scale the magnetic field in units of $D/g\mu_{\mathrm{B}}$, and explore the effect of changing both the sign and value of the ratio E/D.

The ISC spin-selection rules that determine the coupling coefficients $(m_i, m_{i'})$ follow from the spin-orbit allowed transitions indicated in the corresponding Jablonski diagrams (Supplementary Figure 10 and Supplementary Figure 13). In cases where no spin-orbit transition is allowed by symmetry, we assume the transition proceeds spin-nonselectively *via* other means, *i.e.*, $p_x = p_y = p_z = \frac{1}{3}$. We also explore the effect of relaxing the predicted spin-selectivity in some cases, e.g. setting an $|s_x\rangle$-selective transition to have $p_x = 1 - 2\epsilon$, while $p_y = p_z = \epsilon$, as a function of a small parameter $\epsilon \ll 1$.

The rates Γ_s, Γ_e, Γ_{ISC1}, and Γ_{ISC2} can be estimated from an analytical three-level model for the autocorrelation function [9]. Full fluorescence saturation curves were not recorded for fear of photobleaching the emitters of interest, so we introduce a free parameter to quantify the relative saturation of the optical dipole transition, $x = \Gamma_e/\Gamma_s$. We expect that $x \approx 0.1$–0.5 based on partial saturation curves. In this case, and assuming $\Gamma_{\mathrm{ISC2}} \ll \Gamma_{\mathrm{ISC1}}$ (as justified by later analysis), a three-level model yields approximate analytic expressions for the underlying rates as a function of the observed antibunching (τ_1) and bunching (τ_2) timescales and the bunching amplitude (C_2) in the fluorescence autocorrelation function, which takes the general form:

$$g^2(t) = 1 - C_1 e^{-t/\tau_1} + C_2 e^{-t/\tau_2}, \tag{13}$$

where

$$\Gamma_s \approx \frac{1}{\tau_1}\left(\frac{1}{1+x}\right), \tag{14a}$$

$$\Gamma_{\text{ISC2}} \approx \frac{1}{\tau_2}\left(\frac{1}{1+C_2}\right), \tag{14b}$$

$$\Gamma_{\text{ISC1}} \approx \frac{1+x}{x}\left(\frac{1}{\tau_2} - \Gamma_{\text{ISC2}}\right). \tag{14c}$$

Of these values, only Γ_{ISC1} varies strongly with x in the expected range $x \approx 0.1$–0.5. This implies that the true value of Γ_{ISC1} remains uncertain based on our measurements, but nevertheless the simulations are robust to variations in x when parameterized in this way. For example, the average values of the autocorrelation parameters based on the zero-field measurements shown in Fig. 3 of the main text are (see Supplementary Table 1):

$\tau_1 = 1.1$ ns, $\tau_2 = 1.4$ s, $C = 5.4$

Assuming $x = 0.5$, this implies that

$\Gamma_s \approx 600$ MHz, $\Gamma_{\text{ISC1}} \approx 1.8$ MHz, $\Gamma_{\text{ISC2}} \approx 0.11$ MHz

II. SUPPLEMENTARY DISCUSSION

A. PL Simulations

We performed calculations using this model for all of the level structures described in the previous section. Simulations of the PL as a function of in-plane magnetic field across a range of parameters are shown in §III. Since we lack a concrete estimate for the triplet spin-anisotropy parameter (E/D) and the spin lifetime (T_1), we performed calculations spanning a range of their values.

For the case of a singlet ground-state, there is only one level-structure of interest [Supplementary Figure 10(b)], since Supplementary Figure 10(a) does not include spin-dependent selection rules. The simulations of this system exhibit clear variations of steady-state PL as a function of in-plane field, with four bright lobes offset from the x and y axes. The pattern exhibits near fourfold symmetry especially near $E/D \sim -0.3$, and the contrast between bright and dark regions increases with T_1. The PL exhibits a minimum at $B = 0$, and maintains this minimum value for $\mathbf{B}$ applied along the z axis. As discussed below, this model reproduces many features of our experiments, and is a leading candidate to explain the observations.

The simulations of triplet-ground-state systems (Supplementary Figure 13) generally fall into distinct categories based on the arrangement of ISC selection rules, namely:

- *Class I: No Spin Selection Rules*

  In diagram (a), similar to case (a) of the singlet-GS configuration, no ISC transitions are allowed by spin-orbit coupling. It is possible that ISC transitions can proceed via different means, but they are likely to be spin-nonselective and therefore do not depend on an external magnetic field.

- *Class II: Symmetric ISC involving $|s_x\rangle$ or $|s_y\rangle$*

  In diagrams (b) and (d), both ISC transitions are selective to the same spin projection. This configuration does produce anisotropic changes in PL as a function of field direction, however the modulation amplitude is small when the experimentally-relevant ISC rate parameters are used in simulations (the contrast is only $\sim 0.1\%$ in the simulations of §III). Moreover, the patterns general exhibit 180° rather than 90° symmetry, with a clear difference in brightness for a field oriented along x or y. Finally, the PL is predicted to decrease slightly as a function of increasing B applied along z, in contrast to our experiments.

- *Class III: ISC involving $|s_z\rangle$ only*

  Diagrams (c), (e), and (f) involve $|s_z\rangle$-selective ISC transitions only. Simulations of these level structures exhibit patterns of PL variations broadly similar to the singlet-GS diagram Supplementary Figure 10(b) discussed above, with approximate fourfold symmetry as a function of in-plane field. The triplet-GS patterns are inverted with respect to the singlet-GS case, with a global PL maximum at $B = 0$ and four dark lobes when the field is not aligned with either the x or y axes. The PL is independent of B applied along z (*i.e.*, it remains bright). Diagram (e), consisting of one $|s_z\rangle$-selective ISC transition and one spin-nonselective transition, exhibits a large PL contrast, whereas the contrast for diagrams (c) and (f) is much smaller than we observe in experiments.

- *Class IV: Asymmetric ISC involving $|s_x\rangle$ and $|s_y\rangle$*

  In the case of diagrams (g) and (h), if the upper and lower ISC transitions proceed entirely through the lowest ($^1A_1$) singlet state, the result is a pattern of PL variations as a function of in-plane field orientation with approximate four-fold symmetry and — especially for increasing T_1 — relatively strong contrast. These features generally agree with our observations; however, the simulations also predict that the PL should reach a maximum at $B = 0$ and decrease strongly for B applied along z, in clear contradiction to experiments.

- *Class V: Asymmetric ISC involving $|s_x\rangle$ or $|s_y\rangle$ and $|s_z\rangle$*

  Alternative schemes for diagrams (g) and (h) invoke the ISC from the triplet excited state to the higher-lying singlet state, which is selective for the $|s_z\rangle$ spin projection. Simulations for these schemes exhibit strongly anisotropic PL as a function of in-plane field orientation, but clear 180° symmetry as compared to the 90° symmetry we observe in experiments. A further pattern emerges when comparing simulations for asymmetric ISC level structures in classes IV and V: the PL is generally bright for fields oriented along the two orthogonal directions corresponding to allowed spin transitions but strongly suppressed when the field is applied along the third axis.

On the basis of these simulations, we conclude that configurations in Class III, especially diagram (e), together with the singlet-GS case (b), exhibit the greatest similarity with our experiments. We subsequently investigated the behavior of these two models in greater detail, considering especially the predicted behavior of the photon emission autocorrelation function as a function of magnetic field.

By comparing the simulations of steady-state PL and the photon autocorrelation function to our experimental observations, we arrived at the set of model parameters listed in Supplementary Table 5 that are used for the simulations presented in Figure 4 of the main text. In both cases, we set $E/D = 0.33$, corresponding to the configuration with clearest 90° symmetry for in-plane fields. We also set the saturation parameter $x = 0.1$, which subsequently determines the relative magnitude of Γ_e and Γ_{ISC1} as discussed previously. We confirmed that the results are generally independent of $x \in [0.1, 1]$ as long as other parameters are scaled appropriately.

Note that no quantitative fit or numerical optimization procedure was performed. Rather, we manually adjusted parameters in order to approximate the key observations. We therefore do not make a claim regarding confidence intervals or the uniqueness of this solution. It is clear, in fact, that some parameters exhibit strong covariance with respect to observations (the covariance of the optical excitation rate and the ISC rate Γ_{ISC1} is one such example). Furthermore, various subtle features of the data are not reproduced by our simple model. Our primary goal is to establish the feasibility of simple electronic level structures to explain the main features in experiments and ideally to uncover the leading candidates such that they can guide future measurements and calculations.

Supplementary Figure 16 shows the simulated PL as a function of in-plane field angle together with the total steady-state population in the metastable triplet (singlet) for the case of the singlet (triplet) ground-state model. As expected, the metastable population is inversely correlated with the PL. Supplementary Figure 17 shows the variation of the spin-triplet eigenstates for a fixed field amplitude ($g\mu_{\mathrm{B}}B/D = 0.5$) together with the PL for the singlet-GS case. The triplet Hamiltonian is identical in the triplet-GS case.

Finally, we simulate the photon autocorrelation function for various settings of in-plane field angle, and fit the result to the empirical model discussed in the text, with either two or three rates ($n = 2$ or 3). The simulated curves are

Supplementary Table 5. **Simulation Parameters.**

**Parameter**	**Singlet-GS [model (b)]**	**Triplet-GS [model (e)]**
E/D	-0.33	-0.33
T_1 (μs)	50	50
Γ_s (MHz)	820	820
Γ_e (MHz)	82	82
Γ_{ISC1} (MHz)	7.7	33
Γ_{ISC2} (MHz)	0.85	0.13
ϵ^*	0.02	0.05

*The parameter ϵ relaxes the spin selectivity of the spin-orbit-allowed transition as described in the text.

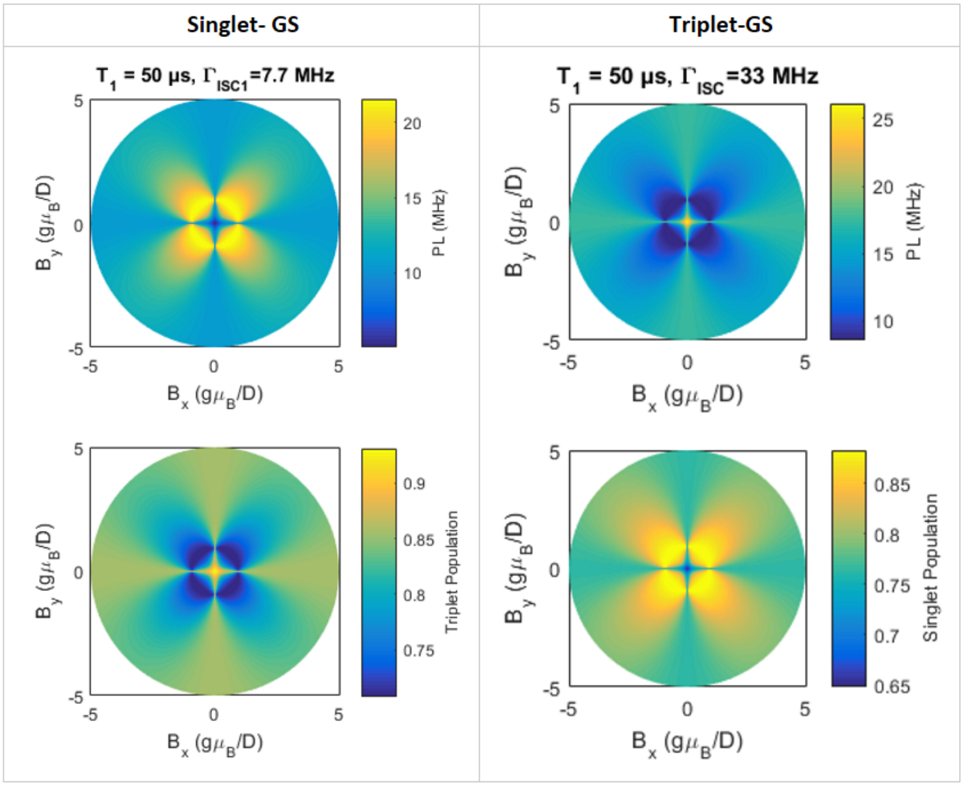

Supplementary Figure 16. **PL Simulations.** Steady-state PL as a function of in-plane magnetic field (top panels) as well as the steady-state population in the metastable state (bottom panels).

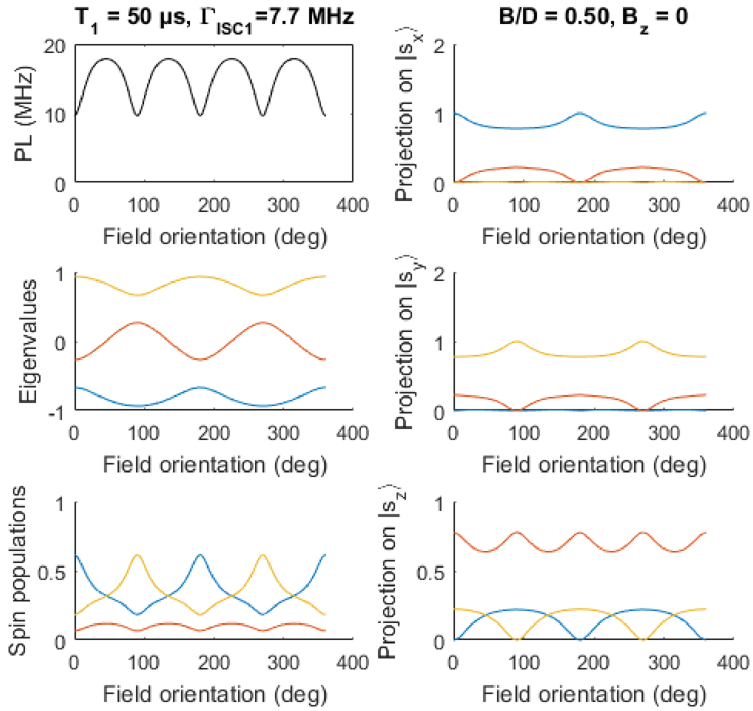

Supplementary Figure 17. **Spin Simulations.** Steady-state PL as a function of in-plane magnetic field orientation (top left) for the singlet-GS model together with the eigenvalues, steady-state populations, and projections of the metastable-triplet spin eigenstates.

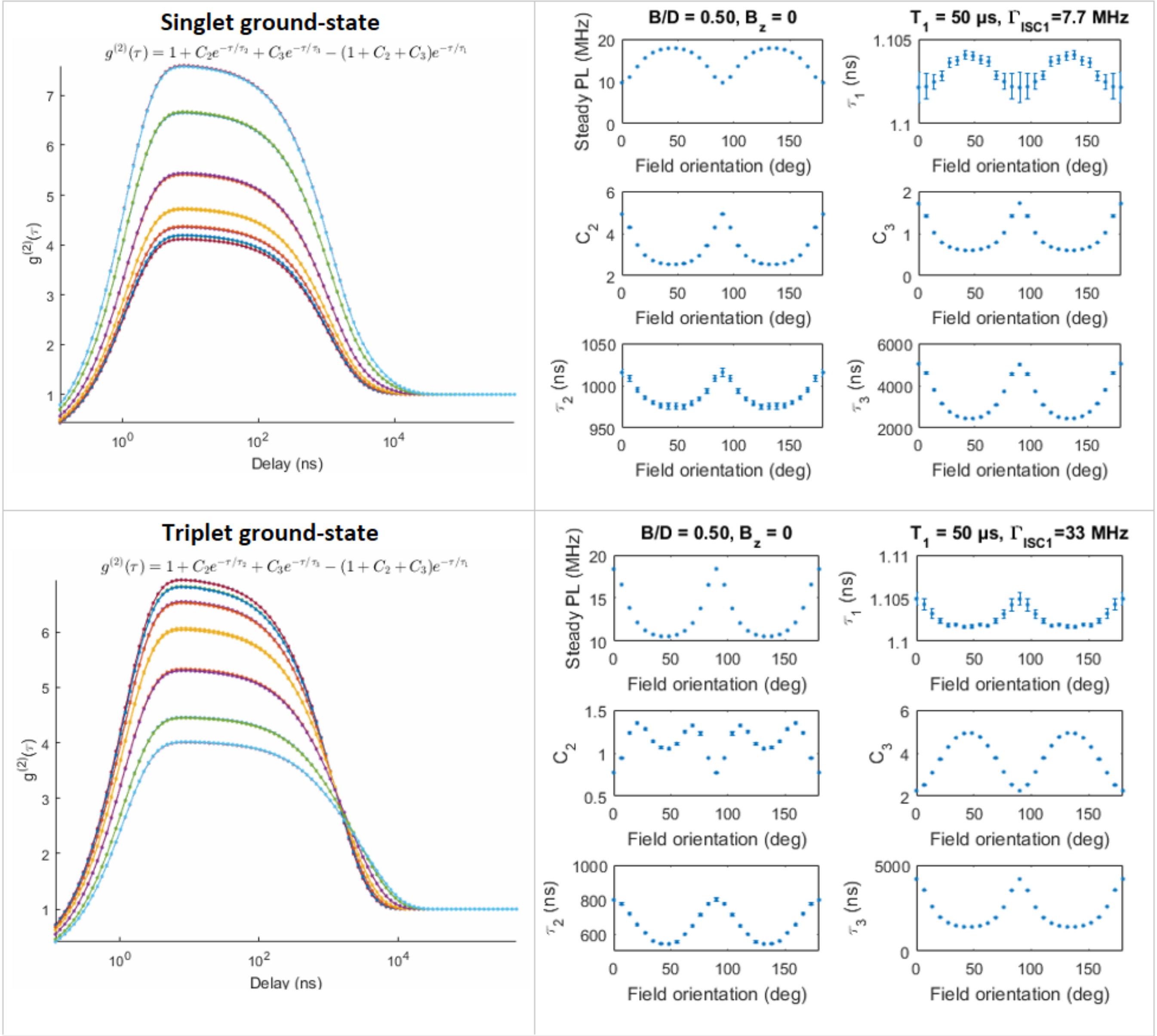

Supplementary Figure 18. **Autocorrelation Simulations.** Left panels: Simulated photon autocorrelation function for different orientations of an in-plane magnetic field. Right panels: Best-fit parameters from a three-rate empirical model.

shown in Supplementary Figure 18 along with three-rate fits. The corresponding results of two-rate fits are plotted in Fig. 4 of the main text.

In comparing these models, we observe the following qualitative differences that lead to our conclusion that the singlet-GS model is the best match to our experimental observation

- The singlet-GS model exhibits dark PL at $B = 0$ and for B oriented along x, y, or z, whereas the PL response in the triplet-GS case is inverted. In our observations the PL is at a minimum when the field is aligned or orthogonal to the observed emission dipole axis, in agreement with the singlet-GS case. Note that the observed excitation dipole is rotated from the emission dipole by $\sim 53^\circ$, in seeming agreement with the triplet-GS case. However, we expect that the emission dipole is a truer representation of the defect's symmetry axis on the basis of its higher visibility and the likely possibility of off-resonant excitation addressing additional short-lived excited states.

- Although both models exhibit similar steady-state PL contrast as a function of in-plane field angle, the corresponding optical dynamics exhibited by the photon autocorrelation function is qualitatively different. In the

singlet-GS case, the bunching lifetime stays nearly constant whereas the amplitude changes strongly, in agreement with our observations. On the other hand, the triplet-GS model exhibits strong variations in both the amplitude and lifetime of bunching. Qualitatively, this makes sense since the bunching lifetime in the strong-excitation regime considered here is related to the effective ISC rate (averaged across all spin levels) from the excited state to the metastable state, whereas the bunching amplitude reflects both ISC rates. In the singlet-GS case, the excited-state ISC is independent of field whereas the ground-state ISC varies strongly, and vice versa for the triplet-GS case. The clear difference in photon emission statistics is strong evidence in favor of the singlet-GS case as a model for our observations.

Nonetheless, some features of the experiments are not reproduced by either simulation. For example, the autocorrelation function in the singlet-GS case in the presence of an in-plane field is better described by a three-rate model, compared to the two-rate shape observed in experiments. Furthermore, neither model predicts the increase in PL we observe as a function of B applied along z or the non-monotonic changes at small values of B applied along x or y. We hypothesize that these discrepancies might be related to other physical effects such as hyperfine or spin-orbit coupling between levels that are not currently included in our model. Hyperfine coupling is certain to play some role due to the high nuclear spin density in h-BN. Alternatively, perturbations due to strain or local electric fields could lower the defect's symmetry or rotate its coordinate axes relative to h-BN's crystal orientation. Further experiments are required to fully answer these and other questions raised by this work. In the next section we explore possible hyperfine interactions and their effects.

B. Hyperfine interactions

Hyperfine interactions in hBN are expected to be complex because multiple nuclear spins will couple to any defect electron spin due to every atomic site possessing a nuclear spin. Such complicated interactions have been observed in electron paramagnetic resonance (EPR) spectroscopy of bulk hBN [10, 11]. The size of the observed hyperfine structure was on the order of 30 MHz. However, much larger interactions (up to ≈ 1 GHz) are theoretically possible, as we describe in the subsection below. Also below, we estimate the magnitude of the electron ZFS parameters to be 1–6 GHz.

If the hyperfine and ZFS parameters are of a similar size, then the hyperfine interactions may explain the features currently not captured by our purely electronic optical dynamics model: (1) the increase in the PL with the application of a magnetic field along z that saturates after ≈ 200 G, and (2) the non-monotonic changes at small values of magnetic fields applied along x or y with turning points at ≈ 70 G. A crude explanation is that the hyperfine interactions define an effective in-plane internal magnetic field that is experienced by the electron spin. Only when this internal field is overcome by the applied field does our purely electronic modeling apply. This would explain (1) and (2) and indicate that the internal fields correspond to hyperfine interactions of the order of ≈ 560 MHz and ≈ 196 MHz, respectively, which are values reasonably consistent with our estimates below. However, we emphasize that more precise calculations of the effects of hyperfine interactions are required to confirm this explanation and these can only be performed once further information about the defect (*i.e.*, its structure, ZFS parameters, etc.) is established.

1. Estimation of electronic ZFS parameters

Assuming that the ZFS parameters arise from spin-spin interaction between two unpaired electrons of a triplet level, approximate expressions for the parameters are [2]

$$D = \frac{3}{2}\frac{\mu_0}{4\pi}g^2\mu_B^2\langle\frac{1-3x_{12}^2/r_{12}^2}{r_{12}^3}\rangle \tag{15a}$$

$$E = \frac{3}{2}\frac{\mu_0}{4\pi}g^2\mu_B^2\langle\frac{z_{12}^2-y_{12}^2}{r_{12}^5}\rangle \tag{15b}$$

where μ_0 is the vacuum permeability, x_{12}, y_{12}, z_{12} are the coordinates of the displacement vector $\vec{r}_{12}$ with magnitude r_{12} that connects the positions of the two electrons, and the angle brackets denote the direct integral over the unpaired spin density. Note that the exchange integral has been neglected in the above.

Rough estimates of the parameters can be made by assuming that the defect axis is aligned with one of the in-plane bonds and that the spin density is contained in-plane and distributed over next-to-nearest neighbor atoms (*i.e.* as for a vacancy-centered defect). In this case, we can approximate the average separation of the unpaired electrons by the displacement vector between next-to-nearest neighbor lattice sites, such that $|x_{12}| \approx 3l_B/2 = 2.18$Å,

Supplementary Table 6. **Estimated hyperfine parameters of different atomic orbitals in hBN**

π-orbital	$A_\parallel$ (MHz)	$A_\perp$ (MHz)	$\sigma-$orbital	$A_\parallel$ (MHz)	$A_\perp$ (MHz)
^{11}B($I = 1/2$)	64	-127	^{11}B($I = 1/2$)	891	764
^{14}N($I = 1$)	56	-111	^{14}N($I = 1$)	641	530

$|y_{12}| \approx \sqrt{3}l_B/2 = 1.26$Å and $|z_{12}| \approx 0$, where $l_B = 1.45$Å is the bond length of hBN. Using these values, we find $D \approx -6$ GHz and $E \approx -1$ GHz. The relative sign of E and D would change if the spin density was rather primarily contained out-of-plane (*i.e.* in $\pi-$orbitals).

2. Estimation of hyperfine interaction in hBN

The hyperfine interaction between the defect's electronic spin and a given nuclear spin can be described by two parameters

$$A_\parallel = f + d \quad (16a)$$
$$A_\perp = f - 2d \quad (16b)$$

which are defined by the Fermi contact f and dipolar d interactions between the unpaired electron spin density associated with the atom and its nuclear spin [2]. If the unpaired electrons occupy an atomic orbital of the form

$$\psi = c_s\phi_s + c_p\phi_p \quad (17)$$

where ϕ_s and ϕ_p are the $2s$ and $2p$ orbitals, respectively, and c_s and c_p are linear coefficients satisfying the normalization condition $|c_s|^2 + |c_p|^2 = 1$, then the interactions are

$$f = \frac{8\pi}{3}\frac{\mu_0}{4\pi} g\mu_B g_n \mu_n |c_s|^2 \eta |\phi_s(0)|^2 \quad (18a)$$
$$d = \frac{2}{5}\frac{\mu_0}{4\pi} g\mu_B g_n \mu_n |c_p|^2 \eta \langle\phi_p|\frac{1}{r^3}|\phi_p\rangle \quad (18b)$$

where η is the portion of the total spin density at the atom, g_n and μ_n are the nuclear g-factor and magneton, respectively, and r is the distance of the electron spin from the nucleus. The values of $|\phi_s(0)|^2$ and $\langle\phi_p|\frac{1}{r^3}|\phi_p\rangle$ are distinct for B and N and can be obtained from ab initio calculations of the atoms.

For the in-plane sp^2 $\sigma-$orbitals of hBN, the expected values for the linear coefficients are $|c_s|^2 = 1/3$ and $|c_p|^2 = 2/3$, and so these orbitals result in both contact and dipolar interactions. For the out-of-plane p $\pi-$orbitals, the expected values are instead $|c_s|^2 = 0$ and $|c_p|^2 = 1$, and so these orbitals only yield a dipolar interaction. Using the values for $|\phi_s(0)|^2$ and $\langle\phi_p|\frac{1}{r^3}|\phi_p\rangle$ reported by Ref 12 for ^{11}B and ^{14}N (the most prevalent isotopes with spin), we have estimated the hyperfine parameters in Supplementary Table 6 for both $\sigma-$ and $\pi-$orbitals.

The values in Supplementary Table 6 correspond to the hyperfine interactions that would result if all of the electron spin density occupied a single atomic orbital (*i.e.*, $\eta = 1$). In reality, the density will be spread over multiple atomic orbitals. Since at least three atomic orbitals are likely to contribute, we can estimate an upper bound of the hyperfine interactions by dividing the above by 3. This will yield interactions on the order of ≈30 MHz for $\pi-$orbitals and ≈300 MHz for $\sigma-$orbitals.

C. ODMR Simulations

A crucial next step in the study and application of h-BN's spin defects is to attempt ODMR spectroscopy and time-domain spin control. In order to inform these future measurements, we use our master-equation model to estimate the predicted ODMR response as a function of applied dc magnetic field. These estimates highlight the optimal conditions for experiments, and further inform the technological potential of these defects for quantum information processing and quantum sensing.

To simulate the ODMR response within our model, we add additional spin-mixing terms within the triplet manifold. For example, in the singlet-GS model of Supplementary Figure 14, also used in Fig. 4 of the main text, states

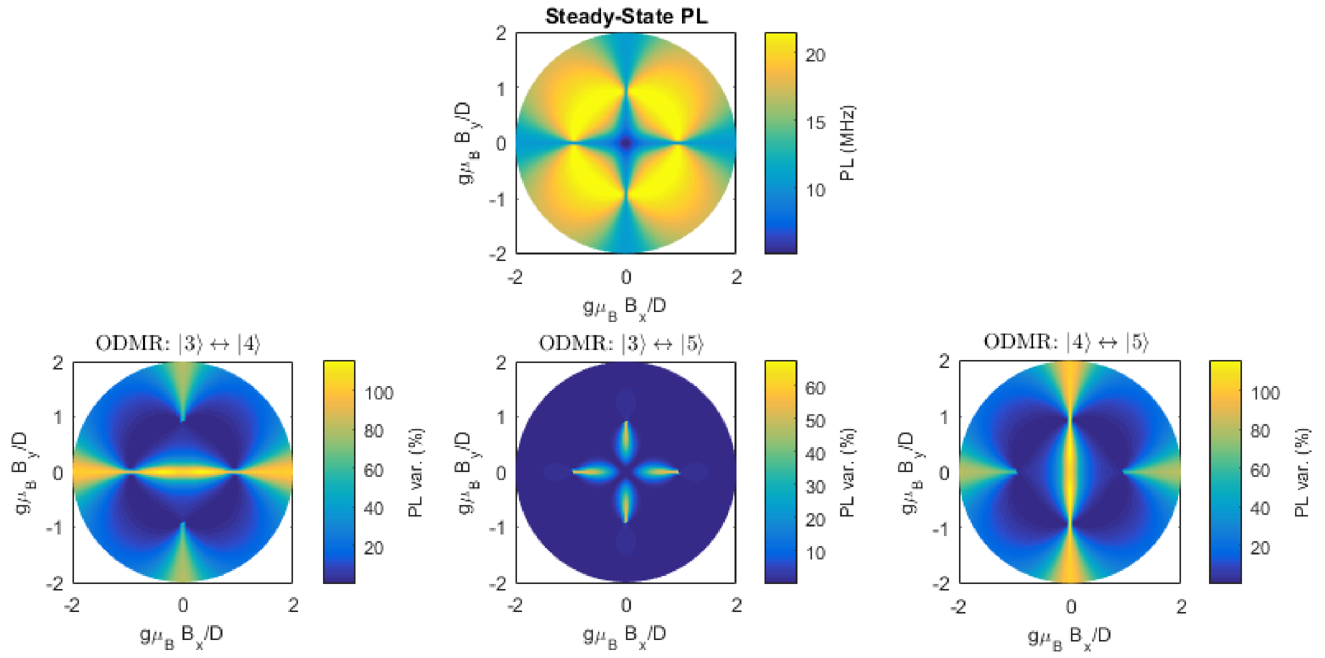

Supplementary Figure 19. **ODMR Simulations.** Predicted ODMR PL variation (bottom panels) as a function of in-plane magnetic field when any two of the triplet eigenstates are resonantly driven by an ac magnetic field. The corresponding steady-state PL is shown in the top panel.

$\{|3\rangle, |4\rangle, |5\rangle\}$ are the triplet eigenstates for the given settings of applied dc magnetic field, and we need only include an additional rate $\Gamma_{ij}^{\text{ODMR}} \gg \Gamma_{\text{ISC2}}$ in order to ensure complete mixing of the states $|i\rangle$ and $|j\rangle$.

Simulations of the ODMR PL variation, *i.e.*, $(I_{\text{MR}} - I_0)/I_0$ where I_{MR} (I_0) is the PL in the presence (absence) of a resonant driving field, are shown in Supplementary Figure 19 for the singlet-GS model (b). The other parameters in the simulation remain those listed in Supplementary Table 5. As expected, the effect of a resonant field that mixes the triplet states is to reduce the steady-state population trapped in the triplet, causing an increase in PL. The effect is most pronounced when the steady-state PL is near a minimum, especially near zero field. The PL variation is also strongest when transitions are driven between states $|3\rangle \leftrightarrow |4\rangle$ or $|4\rangle \leftrightarrow |5\rangle$. This makes sense since state $|4\rangle$ has the largest projection on $|s_z\rangle$ for this range of field settings, and the lower ISC decay from the triplet selects for the $|s_z\rangle$ projection in this level diagram.

Here we are assuming that the ac field direction and magnitude can be chosen to strongly drive the selected pair of triplet eigenstates (we used a fixed value of $\Gamma^{\text{ODMR}} = 100$ MHz to calculate the PL variation). Based on the spin-triplet Hamiltonian, we see that in this case the optimum transitions between state $|4\rangle \sim |s_z\rangle$ and states $\{|3\rangle, |5\rangle\} \sim \{|s_x\rangle, |s_y\rangle\}$ will be driven by in-plane fields oriented along $\hat{y}$ and $\hat{x}$, respectively. In the case of the strong driving fields adopted in the simulations, the ODMR linewidth will be power broadened. Assuming the spin lifetime is limited by the decay of the metastable triplet state, estimated in the simulations to occur with a rate $\Gamma_{\text{ISC2}} = 0.85$ MHz, the minimum ODMR linewidth would be $\approx \Gamma_{\text{ISC2}}/2\pi = 140$ kHz.

III. SUPPLEMENTARY NOTE 1

In Supplementary Figure 20 to Supplementary Figure 30 below we plot simulations of the PL as a function of in-plane magnetic field vector, for various settings of the spin-triplet parameters E/D and T_1. In each case we set the following parameter values based on the analytical three-level model: $\Gamma_s = 600$ MHz, $\Gamma_e = 300$ MHz, $\Gamma_{\mathrm{ISC1}} = 1.8$ MHz, $\Gamma_{\mathrm{ISC2}} = 0.11$ MHz

Singlet Ground-State Models:

Here only level structure (b) is of interest in Supplementary Figure 10, since level structure (a), with no spin-dependent selection rules, does not result in field-dependent PL. Simulations for diagram (b) are shown in Supplementary Figure 20

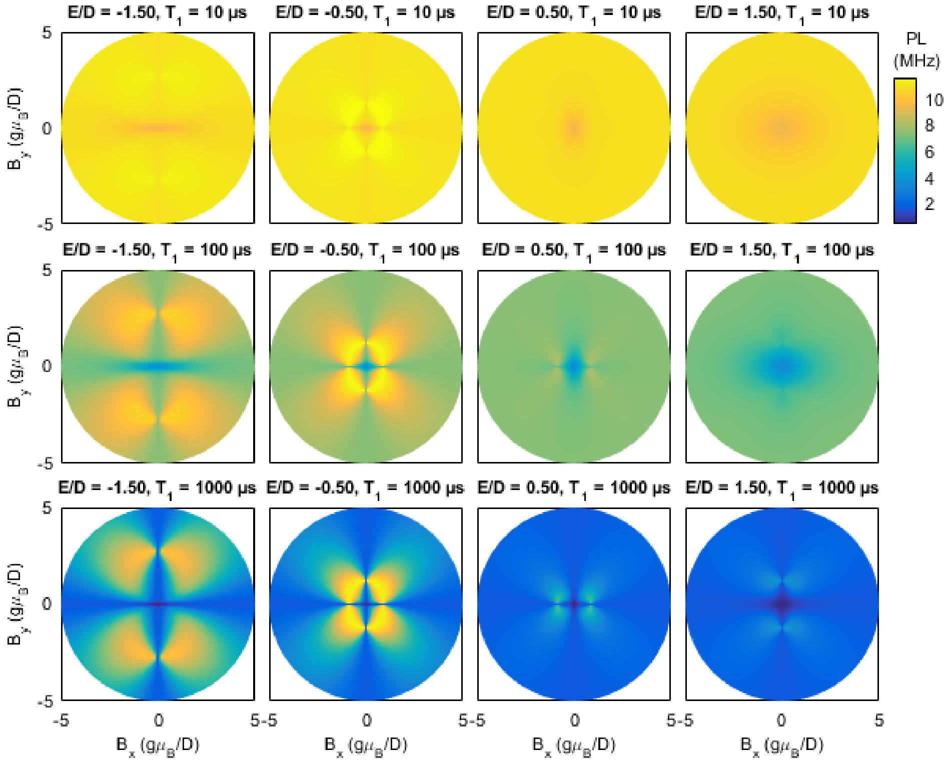

Supplementary Figure 20. PL as a function of in-plane magnetic field for singlet-GS level diagram (b), assuming coupling coefficients $\boldsymbol{m}' = (\frac{1}{3}, \frac{1}{3}, \frac{1}{3})$ and $\boldsymbol{m} = (0, 0, 1)$.

Triplet Ground-State Models:

Level structure (a), with no spin-dependent selection rules, does not produce field-dependent PL. Calculations for the other possible level arrangements are shown below. Unless otherwise noted, we assume that ISC transitions with no spin-orbit-allowed selection rule can proceed via other mechanisms with no spin selectivity.

For level structures (g) and (h), the potential availability of multiple singlet states between the ground- and excited-state triplet levels can be approximated within our model by varying the excited-state coupling coefficients, $\boldsymbol{m}'$, so we include multiple simulations for these diagrams. We also assume any singlet-to-singlet relaxation happens quickly compared to the metastable lifetime and therefore the ground-state ISC coupling coefficients, $\boldsymbol{m}$, are determined solely by the ground-state singlet.

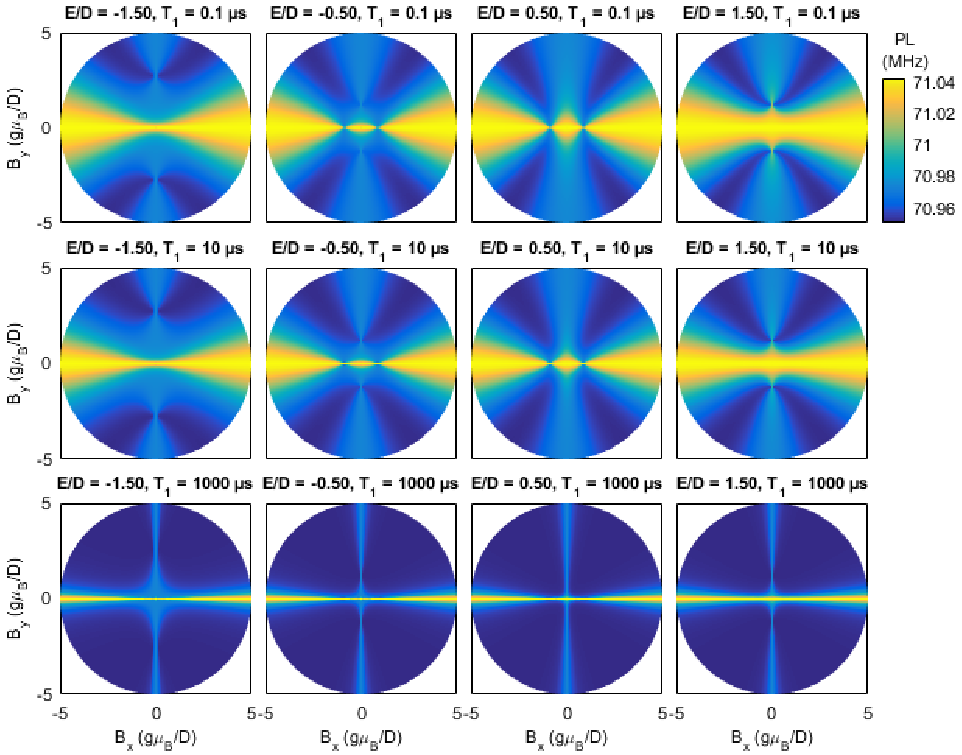

Supplementary Figure 21. PL as a function of in-plane magnetic field for triplet-GS level diagram (b), assuming coupling coefficients $\boldsymbol{m}' = (1, 0, 0)$ and $\boldsymbol{m} = (1, 0, 0)$.

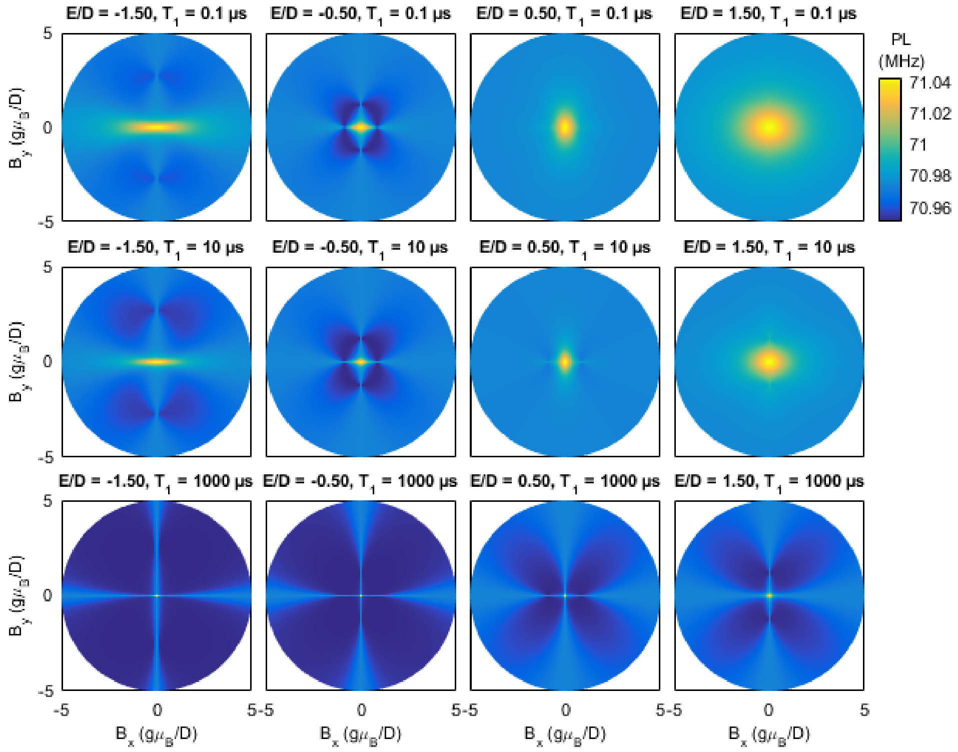

Supplementary Figure 22. PL as a function of in-plane magnetic field for triplet-GS level diagram (c) or (f), assuming coupling coefficients $\boldsymbol{m}' = (0, 0, 1)$ and $\boldsymbol{m} = (0, 0, 1)$.

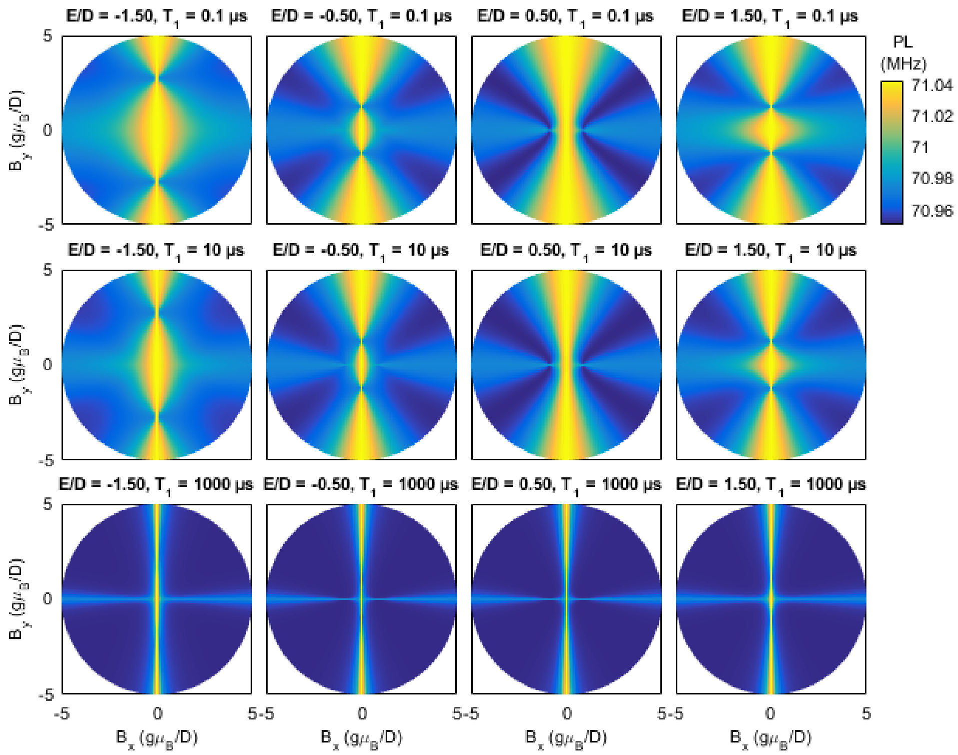

Supplementary Figure 23. PL as a function of in-plane magnetic field for triplet-GS level diagram (d), assuming coupling coefficients $\boldsymbol{m}' = (0, 1, 0)$ and $\boldsymbol{m} = (0, 1, 0)$.

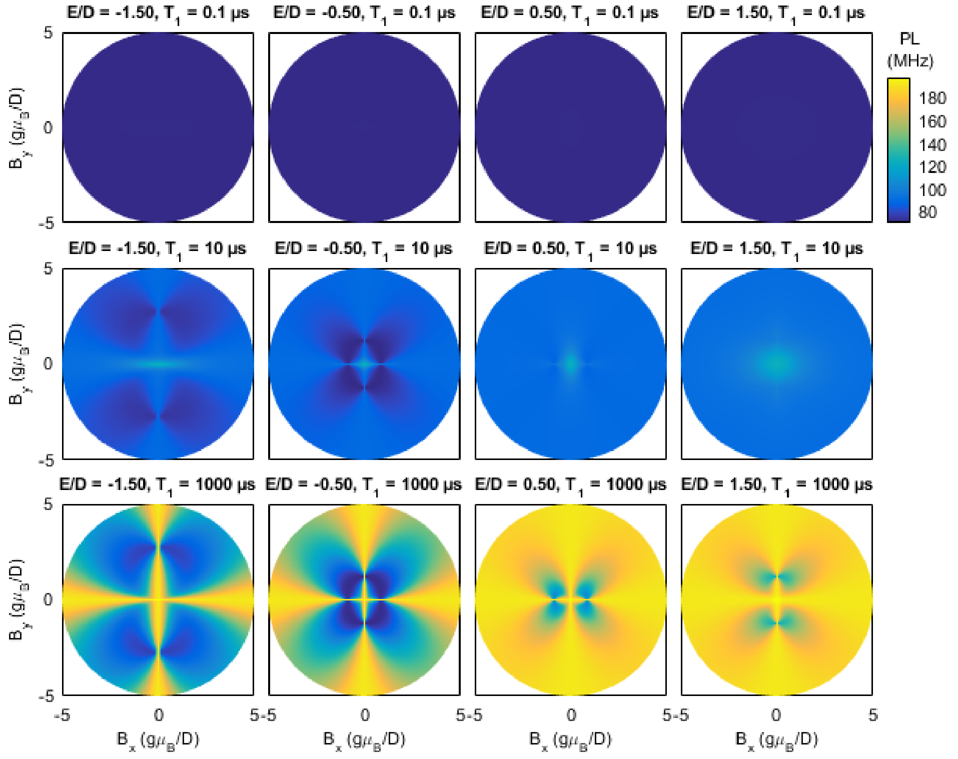

Supplementary Figure 24. PL as a function of in-plane magnetic field for triplet-GS level diagram (e), assuming coupling coefficients $\boldsymbol{m}' = (0, 0, 1)$ and $\boldsymbol{m} = (\frac{1}{3}, \frac{1}{3}, \frac{1}{3})$.

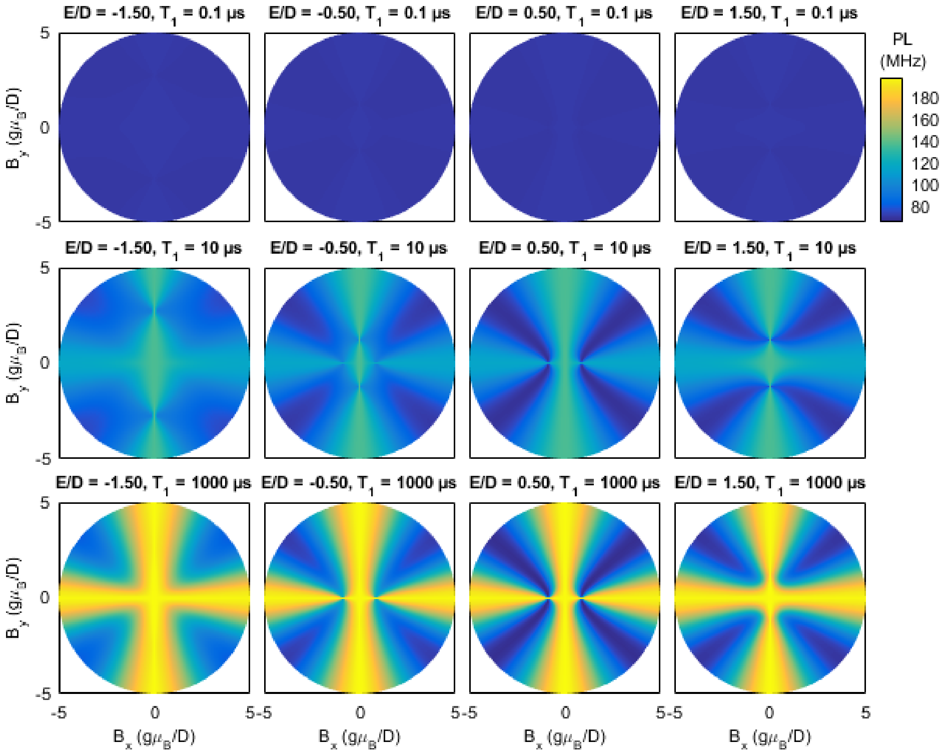

Supplementary Figure 25. PL as a function of in-plane magnetic field for triplet-GS level diagram (g), assuming coupling coefficients $\boldsymbol{m}' = (0, 1, 0)$ and $\boldsymbol{m} = (1, 0, 0)$.

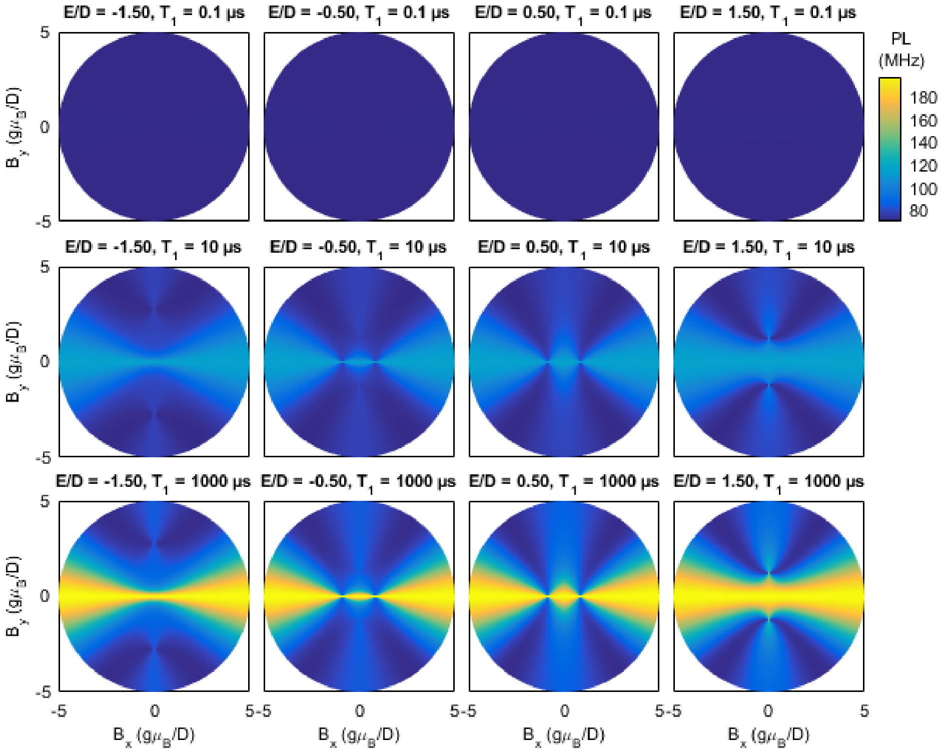

Supplementary Figure 26. PL as a function of in-plane magnetic field for triplet-GS level diagram (g), assuming coupling coefficients $\boldsymbol{m}' = (0, \frac{1}{2}, \frac{1}{2})$ and $\boldsymbol{m} = (1, 0, 0)$.

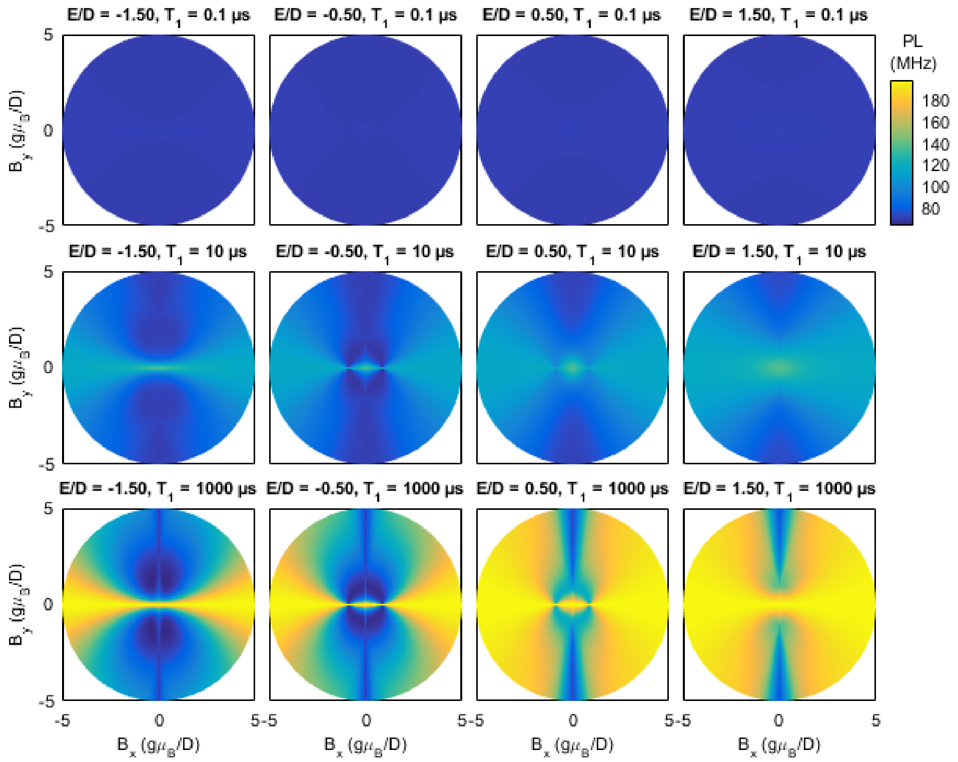

Supplementary Figure 27. PL as a function of in-plane magnetic field for triplet-GS level diagram (g), assuming coupling coefficients $\boldsymbol{m}' = (0, 0, 1)$ and $\boldsymbol{m} = (1, 0, 0)$.

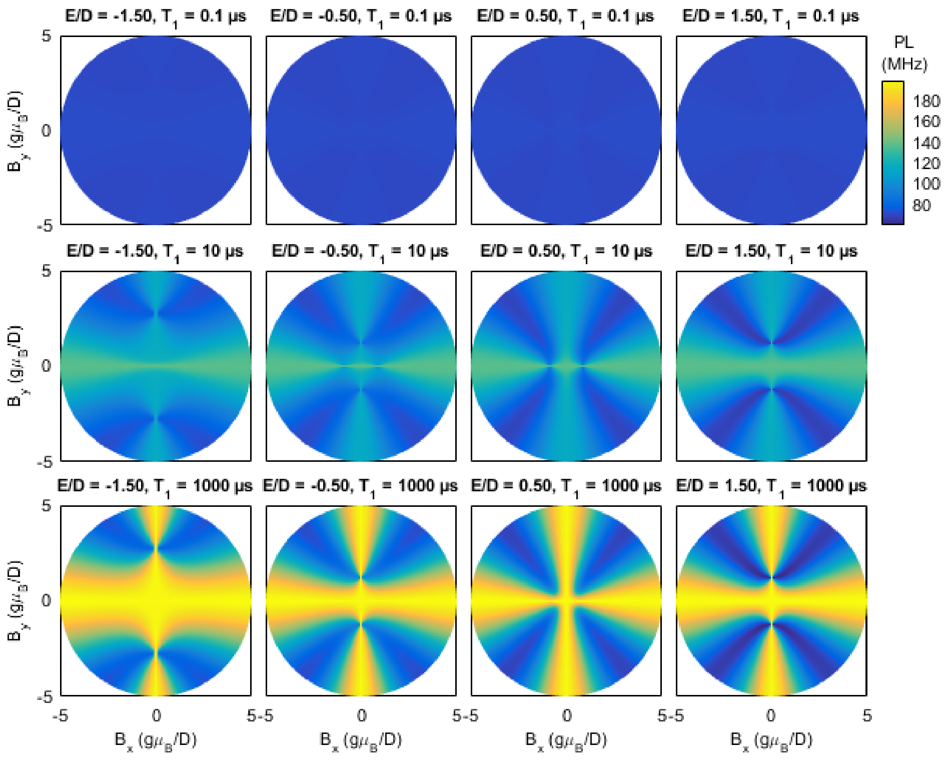

Supplementary Figure 28. PL as a function of in-plane magnetic field for triplet-GS level diagram (h), assuming coupling coefficients $\boldsymbol{m}' = (1, 0, 0)$ and $\boldsymbol{m} = (0, 1, 0)$.

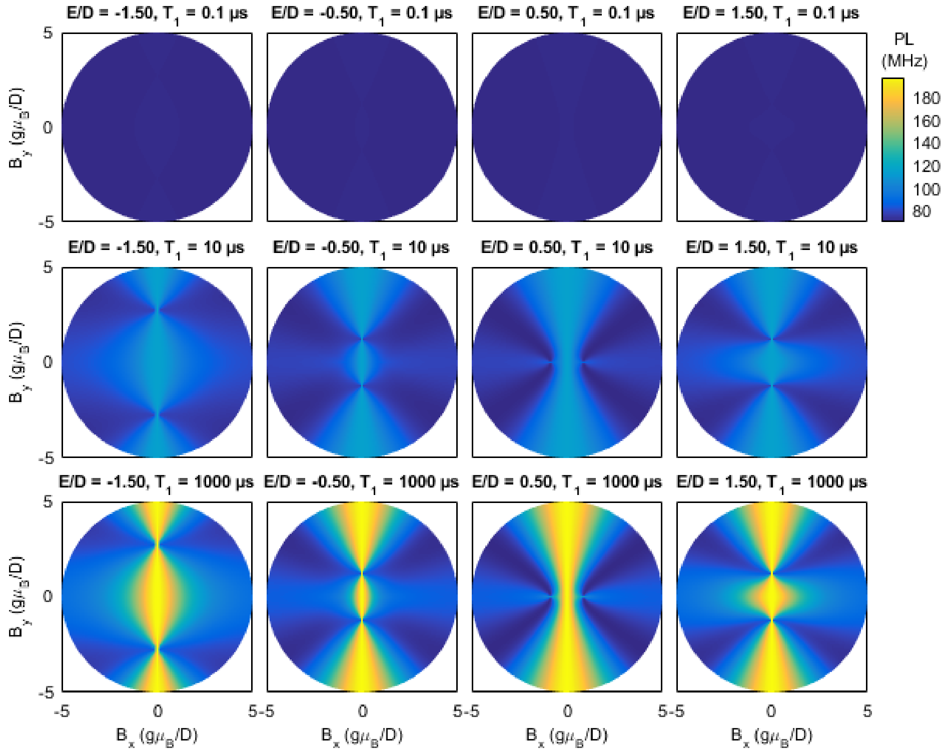

Supplementary Figure 29. PL as a function of in-plane magnetic field for triplet-GS level diagram (h), assuming coupling coefficients $\boldsymbol{m}' = (\frac{1}{2}, 0, \frac{1}{2})$ and $\boldsymbol{m} = (0, 1, 0)$.

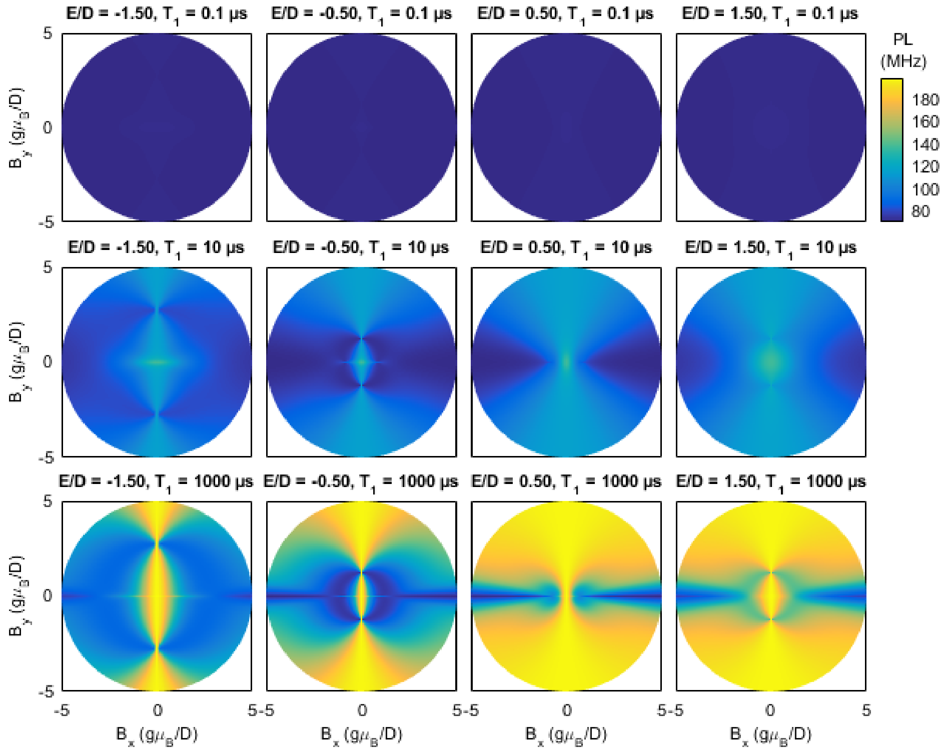

Supplementary Figure 30. PL as a function of in-plane magnetic field for triplet-GS level diagram (h), assuming coupling coefficients $\boldsymbol{m}' = (0, 0, 1)$ and $\boldsymbol{m} = (0, 1, 0)$.

IV. SUPPLEMENTARY REFERENCES

[1] Exarhos, A. L., Hopper, D. A., Grote, R. R., Alkauskas, A. & Bassett, L. C. Optical signatures of quantum emitters in suspended hexagonal boron nitride. *ACS Nano* **11**, 3328–3336 (2017).
[2] Stoneham, A. M. *Theory of Defects in Solids: Electronic Structure of Defects in Insulators and Semiconductors* (Oxford University Press, 1975).
[3] Maze, J. R. *et al.* Properties of nitrogen-vacancy centers in diamond: The group theoretic approach. *New J. Phys.* **13**, 025025 (2011).
[4] Doherty, M. W., Manson, N. B., Delaney, P. & Hollenberg, L. C. L. The negatively charged nitrogen-vacancy centre in diamond: The electronic solution. *New J. Phys.* **13**, 025019 (2011).
[5] Abdi, M., Chou, J.-P., Gali, A. & Plenio, M. B. Color centers in hexagonal boron nitride monolayers: A group theory and ab initio analysis. *ACS Photonics* **5**, 1967–1976 (2018).
[6] Wu, F., Galatas, A., Sundararaman, R., Rocca, D. & Ping, Y. First-principles engineering of charged defects for two-dimensional quantum technologies. *Phys. Rev. Mater.* **1**, 071001 (2017).
[7] Tawfik, S. A. *et al.* First-principles investigation of quantum emission from hBN defects. *Nanoscale* **9**, 13575–13582 (2017).
[8] Koster, G. F., Dimmock, J. O., Wheeler, R. G. & Statz, H. *Properties of the thirty-two point groups* (MIT Press, 1963).
[9] Basché, T., Kummer, S. & Bräuchle, C. *Single-Molecule Optical Detection, Imaging and Spectroscopy*, chap. 2: Excitation and Emission Spectroscopy and Quantum Optical Measurements (VCH Verlagsgesellschaft mbH, Weinheim, Germany, 1996).
[10] Geist, D. & Rmelt, G. Paramagnetische elektronenresonanz in bornitrid. *Solid State Commun.* **2**, 149 (1964).
[11] Fanciulli, M. Electron paramagnetic resonance and relaxation in BN and BN: C. *Philos. Mag. Part B* **76**, 363–381 (1997).
[12] Morton, J. R. & Preston, K. F. Atomic parameters for paramagnetic resonance data. *J. Magn. Reson.* **30**, 577 – 582 (1978).
